# Immediate effects of alcohol marketing communications and media portrayals on consumption and cognition: a systematic review and meta-analysis of experimental studies

**DOI:** 10.1186/s12889-016-3116-8

**Published:** 2016-06-09

**Authors:** Kaidy Stautz, Kyle G. Brown, Sarah E. King, Ian Shemilt, Theresa M. Marteau

**Affiliations:** Behaviour and Health Research Unit, University of Cambridge, Cambridge, UK

## Abstract

**Background:**

Restricting marketing of alcoholic products is purported to be a cost-effective intervention to reduce alcohol consumption. The strength of evidence supporting this claim is contested. This systematic review aimed to assess immediate effects of exposure to alcohol marketing on alcoholic beverage consumption and related cognitions.

**Methods:**

Electronic searches of nine databases, supplemented with reference list searches and forward citation tracking, were used to identify randomised, experimental studies assessing immediate effects of exposure to alcohol marketing communications on objective alcohol consumption (primary outcome), explicit or implicit alcohol-related cognitions, or selection without purchasing (secondary outcomes). Study limitations were assessed using the Cochrane Risk of Bias tool. Random and fixed effects meta-analyses were conducted to estimate effect sizes.

**Results:**

Twenty four studies met the eligibility criteria. A meta-analysis integrating seven studies (758 participants, all students) found that viewing alcohol advertisements increased immediate alcohol consumption relative to viewing non-alcohol advertisements (SMD = 0.20, 95 % CI = 0.05, 0.34). A meta-analysis integrating six studies (631 participants, all students) did not find that viewing alcohol portrayals in television programmes or films increased consumption (SMD = 0.16, 95 % CI = −0.05, 0.37). Meta-analyses of secondary outcome data found that exposure to alcohol portrayals increased explicit alcohol-related cognitions, but did not find that exposure to alcohol advertisements influenced explicit or implicit alcohol-related cognitions. Confidence in results is diminished by underpowered analyses and unclear risk of bias.

**Conclusions:**

Viewing alcohol advertisements (but not alcohol portrayals) may increase immediate alcohol consumption by small amounts, equivalent to between 0.39 and 2.67 alcohol units for males and between 0.25 and 1.69 units for females. The generalizability of this finding beyond students and to other marketing channels remains to be established.

**Electronic supplementary material:**

The online version of this article (doi:10.1186/s12889-016-3116-8) contains supplementary material, which is available to authorized users.

## Background

Alcohol marketing is a prominent feature of an ‘alcogenic’ environment - an environment that reflects and promotes a culture of alcohol use [1]. Alcohol marketing communications have been identified as a potential target for public health intervention due to their proposed influence on harmful patterns of alcohol consumption [2, 3]. The alcohol industry’s position is that marketing raises awareness of certain brands or products, but does not cause overall increased consumption [4, 5].

Findings from three published systematic reviews are discordant with the industry’s position [6–8]. These reviews investigated relationships between exposure to various forms of alcohol marketing and alcohol consumption among young people. Their findings were based on syntheses of overlapping but not identical sets of primary studies. Two of the reviews synthesised evidence from longitudinal cohort studies only [6, 7], whilst the third also incorporated evidence from cross-sectional studies [8]. All three concluded that exposure to alcohol marketing has a dose-dependent association with initiation of alcohol use and increased alcohol consumption.

Due to their focus on people below the legal drinking age these reviews did not include experimental studies, in which participants are randomised to be exposed either to alcohol marketing or a control stimulus, with alcohol consumption objectively measured post-exposure. Whilst the authors of one review [7] note that such studies lack ecological validity (i.e. their settings and procedures may not reflect the complex nature of real-world advertising exposure), experimental studies have several advantages. They allow for a high degree of control over marketing exposure and, with successful randomisation, minimise the potential for confounding of effects by unmeasured variables – a limitation of some longitudinal studies as acknowledged by previous review authors [6–8]. Further, objective measurement of alcohol consumption has benefits over self-report measures used in longitudinal studies, which are prone to influence by participant and contextual characteristics [9]. In synthesising observational or experimental research, there is therefore a trade-off between greater ecological validity and greater internal validity with reduced risk of bias. We are unaware of any systematic attempt to date to synthesise the results of experimental studies on this topic. We therefore conducted the systematic review reported here, to assess evidence from randomised, experimental studies for the immediate effects of exposure to alcohol marketing communications on alcohol consumption, and on alcohol-related cognitions.

Synthesising experimental evidence for the immediate effects of alcohol marketing on consumption to address the limitations of observational longitudinal evidence presupposes that immediate and distal effects of alcohol marketing are related. Alcohol marketing has been hypothesised to promote consumption by normalizing alcohol use and highlighting desirable consequences of consumption [10]. Exposure to marketing is thought to stimulate a motivation to consume alcohol via two sets of processes, conscious and non-conscious. The conscious (explicit) processes include making attitudes to alcohol more favourable and increasing positive expectancies of its use [11–14]. Non-conscious (implicit) processes include priming, imitation, and associative learning, through which general approach orientations towards alcohol are activated [15]. If these causal theories are correct, any immediate and distal effects of exposure to alcohol marketing on consumption are realised through related, though possibly distinct, psychological processes, with positive alcohol-related cognitions being activated immediately in response to a single exposure, as well as developing over time in response to repeated exposures.

Nevertheless, it is important to note that there are myriad other effects of exposure to alcohol marketing not captured by this theoretical framework. Evidence from the marketing literature shows that whilst the objective of any marketing campaign is to increase sales, individual marketing communications have a low likelihood of stimulating immediate urges to purchase and consume the marketed product [16]. Communications are therefore designed to meet additional intermediate objectives such as raising awareness, interest, and identification with products and brands, associating products with certain emotions and experiences, and increasing the number of contexts in which use of the products is seen as appropriate [17, 18]. These outcomes are subtle in nature and develop gradually, meaning they are unlikely to be observed in studies investigating only the immediate effects of marketing exposure.

Marketing campaigns are also typically targeted at distinct demographic subgroups. Early research into alcohol marketing effectiveness conducted by Anheuser Busch, for example, aimed to increase the potency of advertising by linking products with the personality types of consumers [19, 20]. Relatedly, there are individual-level differences in receptivity to specific marketing communications that affect the degree to which a marketing message will be influential. A framework for understanding these differences, the Message Interpretation Process model, highlights that the effectiveness of any marketing message is dependent on audience characteristics such as identification with the characters and messages being presented, and level of scepticism towards the message [13, 21]. Experimental studies of the immediate effects of alcohol marketing do not typically account for factors such as product targeting, identification with brands, and differences in receptivity. Any observed effects on consumption and cognition may therefore be diluted.

With these caveats in mind, the present systematic review and previous reviews can be seen as examining complementary bodies of evidence that have different strengths and limitations, and that each add to understanding of the complex relationship between alcohol marketing and consumption. Consistency between their respective findings would bolster the claim that alcohol marketing has a causal impact on alcohol consumption. However, we acknowledge that even the combination of these two bodies of evidence is still unable to explain the full breadth of the possible public health consequences of alcohol marketing.

To ascertain whether certain population subgroups are more susceptible to the effects of alcohol marketing, it is important to explore whether any immediate effects vary by demographic, behavioural, or cognitive characteristics. For example, alcohol marketing has been found to promote gender stereotypes [22], and as such there may be gender differences in responses to marketing. Also, heavier drinkers have shown attentional bias towards alcohol-related cues [23] and may be more likely to attend to and be influenced by alcohol marketing. Similarly, individuals with weaker executive function or who are more impulsive may be more susceptible to marketing communications [24].

### The present study

The primary objective of this systematic review was to estimate the direction and size of immediate effects of exposure to alcohol marketing communications on alcohol consumption and alcohol-related cognitions, using data collected from individually randomised, experimental studies. The secondary objective was to investigate whether the following factors explain any observed heterogeneity in effects between studies: type of alcoholic drink being marketed or consumed, participant demographic characteristics (gender, age, and level of education), baseline alcohol consumption, and executive function.

## Method

This systematic review was conducted according to Cochrane methodological standards for systematic reviews of health interventions [25] and is reported in line with PRISMA (Preferred Reporting Items for Systematic Reviews and Meta-analyses) guidelines [26]. The review protocol was prospectively registered in the PROSPERO database (ID: CRD42013005057 [27]).

### Search strategy

Electronic searches of seven research literature databases (MEDLINE, EMBASE, PsycInfo, ASSIA, Science Citation Index Expanded, Social Sciences Citation Index, and Econlit) and two grey literature databases (CPCI – Science, and CPCI - Social Science & Humanities) were conducted up to 21 September 2015. Details of electronic searches are provided in Additional file 1. Searches of eligible studies’ reference lists and forward citation tracking (using Google Scholar and PubMed) were also conducted. No restrictions were imposed for publication date, format, or language.

### Inclusion criteria

Individually randomised, laboratory-based experiments with between- or within-participants designs were included. Quasi-experimental and non-experimental studies were excluded. There were no restrictions on types of participants. The eligible intervention was exposure to alcohol marketing communications, defined as any form of advertising, communication or media exposure designed to encourage favourable cognitions towards alcoholic beverages, their purchasing, or consumption. Interventions involving communications negatively valenced towards alcohol (e.g. public health messages) were excluded. The eligible comparator was no exposure to alcohol marketing, or exposure to any other form of alcohol marketing. The primary outcome was objectively measured alcoholic beverage consumption (total amount consumed). Eligible secondary outcomes (proposed mediators of the immediate effect alcohol marketing communications on consumption) were: explicit and implicit alcohol-related cognitions, alcoholic beverage purchasing; and alcoholic beverage selection without purchasing.

### Study selection and data extraction

Full details of procedures for selecting eligible studies and extracting data are presented in the systematic review protocol [27]. Retrieved title-abstract records were independently screened by KB, SK, and KS, with IS acting as arbiter in case of disagreements. Full-text screening of potentially eligible study reports was undertaken using the same procedure. Data on the characteristics of included studies were extracted by one reviewer (KS, KB, or SK). Outcome data were extracted in duplicate by two reviewers (two of KS, KB and SK) working independently, with discrepancies resolved by discussion and IS acting as arbiter when needed.

### Risk of bias assessment

The Cochrane Risk of Bias tool [28] was used to assess potential bias in studies that included the primary outcome, alcohol consumption. Assessments were conducted in duplicate by two reviewers (two of KS, KB and SK) working independently. Unpublished information needed to inform assessments was sought by contacting study authors. Nine domains were considered: random sequence generation, allocation concealment, blinding of participants and personnel, blinding of outcome assessors, incomplete outcome data, selective outcome reporting, baseline comparability between groups, consistency in intervention delivery, and whether objective outcome measures had been assessed for validity and reliability. Risk of bias judgements in three key domains (sequence generation, blinding of participants, and baseline comparability between groups), judged by the authors conducting assessments to be most likely to impact on confidence in study-level estimates of alcohol consumption, were used to determine a summary study-level risk of bias. (Non-) blinding of outcome assessors was judged unlikely to impact on confidence in estimates of this effect, due to the inclusion criterion that consumption was objectively measured. Assessments of incomplete outcome data and selective outcome reporting were also judged unlikely to impact on confidence in estimates; in the former case due to expected low rates of attrition among studies using this specific experimental paradigm (with immediate post-exposure outcome measurement), and in the latter case due to the anticipated lack of study pre-registration for use to reliably assess this dimension.

Reporting biases were assessed by visual inspection of funnel plots. Formal statistical tests to investigate the degree of funnel plot asymmetry [29] were not conducted as planned due to the small number of studies incorporated into each meta-analysis.

### Data synthesis

As the scales used to measure the primary outcome varied between studies, we computed study-level standardised mean differences (SMD) between comparison groups with 95 % confidence intervals. Where outcome data could not be obtained from either a study report or its authors, replacement standard deviations were imputed using established methods [30].

Meta-analyses [31] were conducted using Review Manager 5.3 [32]. For each meta-analysis, we estimated both random effects and fixed effects models and compared results. Statistical heterogeneity was assessed using the *Chi*^*2*^ test and the *I*^*2*^ statistic, and by examining the random effects between study variance (*Tau*^*2*^). Sensitivity analyses were performed to assess the robustness of results to the exclusion of studies with high risk of bias and studies for which we had imputed standard deviations due to missing data.

Subgroup analysis, planned *a priori,* was used to explore gender differences in effects. Other planned subgroup analyses (type of alcoholic drink being marketed or consumed, participant age, education, baseline alcohol consumption, and executive function) could not be conducted due to a lack of measurement and/or reporting of outcome data by subgroups in primary studies. A planned subgroup analysis by media channel could not be conducted as only two media channels, alcohol advertisements and alcohol portrayals in television programmes or films, were investigated among included studies. Effects of exposure to alcohol advertisements and alcohol portrayals were analysed separately.

## Results

### Results of the search

**Fig. 1:**
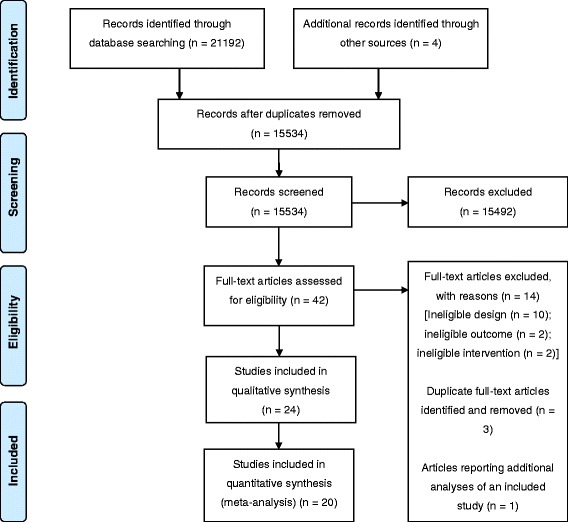
PRISMA flow diagram

Figure 1 illustrates the flow of studies through the systematic review process. Searches yielded a total of 21,192 study records, of which 5,662 were discarded as duplicates. Three additional records were identified through searches of reference lists. One additional record was a recently published paper by members of the research team. All studies screened were reported in English. Title-abstract screening of the 15,534 unique records identified 42 studies judged potentially eligible. Full-text screening resulted in the exclusion of a further 17 studies and the remaining 24 studies (reported in 25 articles, one of which reported a subgroup analysis of another included study [33]) were accepted into the review. Principal reasons for study exclusion are presented in Additional file 2.

### Characteristics of included studies

Tables 1 and 2 show the key characteristics of included studies. Detailed information is presented in Additional file 3. Twenty three of the 24 included studies had been published in peer-reviewed journals and the other [34] was a published dissertation. Eleven studies assessed the primary outcome of alcohol consumption [34–44], and 14 assessed secondary outcomes [40, 45–57], with one study assessing both [40]. Eighteen included studies were conducted in laboratory settings. Of the other six included studies, all assessing secondary outcomes only, four were conducted in a school classroom setting [49, 51, 55, 56], and two online [45, 46]. All but one study had a between-participants design, with the exception using a within-participants design [55]. Participants of included studies were exposed to alcohol advertisements [35–38, 40, 43–45, 47, 49, 50, 53, 55–57], alcohol portrayals in television programmes [34, 42, 43, 48, 51, 54] or films [35, 39, 41, 46, 52], or a combination of both advertisements and portrayals [35, 43]. The advertisements presented were mostly television advertisements, with two exceptions being a study that used magazine advertisements presented as slides [53], and a study that used alcohol advertisements presented within screenshots of the social media website *Facebook* [45]. The portrayals presented tended to show characters consuming alcohol in positive contexts (e.g. celebration) and with positive consequences (e.g. companionship), often in comedy films or programmes. It was not possible to ascertain whether the alcohol portrayals presented were genuine examples of alcohol marketing (e.g. product placement paid for by an alcohol company) or incidental.

**Table 1:** Key characteristics of included studies assessing primary outcome - alcohol consumption

Study	Country	Participants that completed study	Baseline alcohol consumption	Intervention	Comparison	Outcome
Engels et al. (2009) [35]	Netherlands	80 undergraduates; aged 18–29 (M = 21.45, SD = 2.19); 0 % female	Mean of 21.05 alcoholic beverages in past week	Film (*“American Pie 2”* – a comedy) with 41 alcohol portrayals (characters drank alcohol 18 times and alcoholic beverages were portrayed 23 times)	Film (*“40 Days and 40 Nights”* – a comedy) with 18 alcohol portrayals (characters drank alcohol 3 times and alcoholic beverages were portrayed 15 times)	Number of beer bottles consumed during film
				Two alcohol advertisements presented alongside non-alcohol advertisements	Only non-alcohol advertisements presented	
Kohn & Smart (1984) [36]	Canada	125 undergraduates; age not reported; 0 % female	Not reported	90 min televised soccer game with either four or nine alcohol advertisements embedded	90 min of same televised game with nine non-alcohol advertisements	Number of beers consumed during both the game and a following 30 min questionnaire session
Kohn & Smart (1987) [37]	Canada	66 undergraduates; age not reported; 100 % female	Not reported	Soap opera and music programme with either four or nine alcohol advertisements embedded	Same programmes with nine non-alcohol advertisements	Number of glasses of wine consumed during programmes
Koordeman, Anschutz, & Engels (2011a) [38]	Netherlands	184 undergraduates; aged 16–28 (M = 22.0, SD = 3.3); 50 % female	Mean of 9.41 (SD =10.24) drinks in past week	Full film (*“Watchmen”*) preceded by four alcohol advertisements	Same film preceded by four non-alcohol advertisements	Bottles and amount (cl) of alcoholic beverages consumed during film
Koordeman, Anschutz, van Baaren, & Engels (2011b) [39]	Netherlands	244 undergraduates; aged 18–29 (M = 21.0, SD = 2.54); 54 % female	Not reported	60 min of film (*“What Happens In Vegas” –* a romantic comedy) with 565 s of alcohol portrayals (alcoholic beverages in possession of a character or mentioned verbally)	60 min of same film, edited to show no alcohol portrayals	Bottles and amount (cl) of alcoholic beverages consumed during film
Koordeman et al. (2012) [40]	Netherlands	159 undergraduates; aged 18–29 (M = 21.08, SD =2.7); 0 % female	A mean of 15.90 alcoholic beverages in past week	60 min of film (*“Planet Earth”*) with six alcohol advertisements embedded	60 min of same film with five non-alcohol advertisements embedded	Bottles and amount (cl) of alcoholic beverages consumed during film
Koordeman, Anschutz, & Engels (2015) [41]	Netherlands	154 undergraduates; aged 18–30 (M = 21.4, SD = 2.57); 0 % female	A mean of 17.36 alcoholic beverages in past week	60 min of film (*“Get Him To The Greek”* – a comedy) with 490 s of alcohol portrayals (alcoholic beverages in possession of a character or mentioned verbally)	60 min of same film, edited to show no alcohol portrayals	Bottles and amount (cl) of alcoholic beverages consumed during film
Roehrich & Goldman (1995) [42]	USA	80 undergraduates; aged 25–45 (M = 25.25); 100 % female	A mean of 5.58 drinking occasions in the past month with 1 to 2 drinks consumed per occasion	3.5 min of television programme (*“Cheers”* – a sitcom) with alcohol portrayals	3.5 min of television programme (*“Newhart”* – a sitcom) showing no alcohol portrayals	Amount of beer (ml) consumed during a taste test
Sobell et al. (1986) [43]	Canada	96 undergraduates; mean age (SD) = 22.55 (3.7); 0 % female	56 % heavy, 22 % moderate, and 22 % light drinkers	60 min of television programme (*“Dallas”* – a drama) containing alcohol portrayals (including 7 drinking scenes, 2 verbal references to alcohol, and 14 visual references)	60 min of same programme edited to show no alcohol portrayals	Amount of beer (ml) consumed during a taste test
				Twelve advertisements embedded, four of which were for beer		
					Twelve non-alcohol advertisements embedded	
Sumarta (2000) [34]	USA	96 undergraduates; aged 21–39 (M = 22.39, SD = 2.96); 50 % female;	22 % heavy, 29 % moderate, 34 % light, and 15 % infrequent drinkers	3.5 min of television programme (*“Cheers”*) with alcohol portrayals (Stimuli identical to that used in Roehrich & Goldman, 1995)	3.5 min of television programme (*“Newhart”*) showing no alcohol portrayals (Stimuli identical to that used in Roehrich & Goldman, 1995)	Amount of beer (ml) consumed during a taste test
Wilks et al. (1992) [44]	Australia	120 undergraduates; aged 18–20; gender not reported	Light (<20 g of alcoholic beverage consumed per day) or moderate-heavy (>20 g) drinkers (numbers of each not provided)	90 min of television programming with either six or twelve alcohol advertisements, along with non-alcohol advertisements, embedded between programmes	90 min of same programming with only non-alcohol advertisements embedded between programmes	Number of standard alcoholic drinks consumed during viewing

**Table 2:** Key characteristics of included studies assessing secondary outcomes – explicit and implicit alcohol-related cognitions, and alcohol selection without purchasing

Study	Country	Participants that completed study	Baseline alcohol consumption	Intervention	Comparison	Outcome
Alhabash et al. (2015) [45]	USA	379 undergraduates; mean age = 20.58 (SD = 1.52); 57.1 % female	Not reported	Twelve Facebook screenshots, six of which featured advertisements for a happy hour at a local restaurant	Twelve Facebook screenshots, six of which featured advertisements for a local financial institution	Explicit alcohol-related cognitions – Intentions to consume alcohol, assessed with four items including *“seeing this screenshot makes me want to have a drink”* with 7 point response scales ranging from *strongly disagree* to *strongly agree*.
Bahk (1997) [46]	USA	211 undergraduates; mean age = 19.81 (SD = 1.53); 64 % female	Not reported	Film (*“A Star Is Born”* – a musical) with alcohol portrayals, edited to remove scenes showing negative consequences of alcohol consumption	Same film edited to remove portrayals and negative consequences of alcohol consumption	Explicit alcohol-related cognitions - Attitudes assessed by agreement with 15 statements including *“drinking relieves tension”* and *“drinking is a necessary part of celebration”*.
Brown et al. (2015) [47]	UK	373 adults from general population; aged 18–40 (M = 28.03, SD = 5.64); 59.5 % female	A mean of 16.02 alcohol units consumed in past week	Eight advertisements, four of which were for alcoholic beverage products	Eight advertisements for non-alcohol products	Explicit alcohol-related cognitions - Attitudes assessed with two items, both preceded *“I consider drinking alcohol to be…”* with 7 point response scales ranging from *very unpleasant* to *very pleasant*, and *very bad* to *very good*.
						Implicit alcohol-related cognitions – Positive implicit attitudes assessed with alcohol version of the Implicit Association Test [66].
						Alcohol selection without purchasing – Choice of £5 voucher for alcohol-related (pub) or non-alcohol-related (café) outlet.
De Graaf (2013) [48]	Netherlands	108 high school students; Aged 14–17 (M = 15.34, SD = 0.78); 45 % female	Not reported	20 min of television programme (*“Jersey Shore”* – a reality show) with alcohol portrayals showing positive consequences of alcohol consumption (bonding, celebrating)	20 min of same programme presented after outcome measure	Explicit alcohol-related cognitions - Attitudes assessed with 5 items including *“I think drinking beer is…”* or *“I think drinking liquor is…”* with responses ranging from *unpleasant* to *pleasant*.
						Explicit alcohol-related cognitions - Outcome expectancies assessed with by 5 items including *“Drinking alcohol makes you have fun”* with responses ranging from *completely disagree* to *completely agree.*
Dunn & Yniguez (1999) [49]	USA	551 elementary schoolchildren; Mean age (SD) = 10.27 (1.04); 49 % female	Not reported	Five beer advertisements	Five soft drink advertisements	Explicit alcohol-related cognitions - Adjectives rated for how often they are experienced when consuming alcohol. Preference mapping analysis used to identify the frequency with which positive and arousing expectancies were reported and co-occurred.
						Explicit alcohol-related cognitions - First associate expectancy measure also used. Participants asked to respond open-endedly to the phrase *“How do people feel when they drink alcohol?”*
Goodall & Slater (2010) [50]	USA	145 undergraduates	Not reported	Four 30 s alcohol advertisements	Four 30 s non-alcohol advertisements	Explicit alcohol-related cognitions – Attitudes toward beer, liquor/mixed drinks, and wine on a scale ranging from 0 (*extremely unfavourable*) to 10 (*extremely favourable*).
		Age and gender not reported				
						Implicit alcohol-related cognitions - Implicit attitudes assessed with the Alcohol Affective Misattribution Procedure [67].
Koordeman et al. (2012) [40]	Netherlands	159 undergraduates; aged 18–29 (M = 21.08, SD = 2.7); 0 % female	A mean of 15.90 alcoholic beverages in past week	60 min of film (*“Planet Earth”*) with six alcohol advertisements embedded	60 min of same film with five non-alcohol advertisements embedded	Explicit alcohol-related cognitions - Positive expectancies, assessed with a 6-item scale. Participants indicated their level of agreement with the statement: *“Drinking makes me…”*, with items including *fun* and *happy*.
Kotch et al. (1986) [51]	USA	43 elementary schoolchildren; age not reported (19 5^th^ grade, 24 6^th^ grade); 56 % female	Not reported	35 min of television programme (not specified) edited to contain 13 scenes in which characters drank an alcoholic beverage in social contexts without negative consequences	35 min of same television programme edited to show no alcohol portrayals	Explicit alcohol-related cognitions - Outcome expectancies assessed with Subjective Expected Utility Scale [68].
						Explicit alcohol-related cognitions – Assessed with “How Wrong Is It” scale [69]. Participants reported attitudes towards 24 possible outcomes following alcohol use.
Kulick & Rosenberg (2006) [52]	USA	108 undergraduates; mean age (SD) = 18.42; 70 % female	6.5 % heavy drinkers, 85 % moderate, and 8.5 % abstinent	20 min of eight film clips, six of which showed alcohol portrayals with positive outcomes (laughing, singing, dancing, and companionship) and two of which showed no alcohol consumption. Each clip was viewed twice.	24 min of eight film clips showing no alcohol consumption. Each clip was viewed twice.	Explicit alcohol-related cognitions - Outcome expectancies assessed with the Comprehensive Effects of Alcohol Scale [70].
McCarty & Ewing (1983) [53]	USA	112 adults; age not reported; 57 % female	40 % heavy drinkers, 36 % moderate, and 24 % light	Eight photographic slides of magazine alcohol advertisements	Ten photographic slides of magazine non-alcohol advertisements	Alcohol selection without purchasing - Amount of alcohol poured into a mixed drink.
Rychatrik et al. (1983) [54]	USA	75 children selected from outpatient waiting room of paediatric clinic; aged 8–11; Gender not reported	Not reported	5.5 min of television programme (*“M.A.S.H.”* – a comedy drama) with alcohol portrayals showing alcohol consumption in positive contexts (companionship, toasting)	5.5 min of same programme edited to show no alcohol portrayals	Alcohol selection without purchasing - Hypothetical choice of beverage (alcoholic or non-alcoholic) to serve to photographs of different individuals.
Slater et al. (1996) [55]	USA	157 high school students; mean age (SD) = 14.45; 0 % female	Not reported	Beer advertisements with sports content and beer advertisements without sports content	Non-alcohol advertisements	Explicit alcohol-related cognitions - Participants were asked to report their thoughts and feelings towards the advertisements in an open-ended thought-listing procedure. A net score was calculated by subtracting summed negative comments from summed positive comments.
Van Hoof et al. (2009) [56]	Netherlands	223 secondary school students; aged 12–18 (M = 14.9); 60 % female	Not reported	Twelve advertisements, six of which were for alcoholic beverages, embedded within 22 min soap opera. Two advertisements each for beer, spirits, and mixed drinks.	Twelve advertisements, six of which were for lemonade, embedded within 22 min soap opera	Explicit alcohol-related cognitions – Positive and negative expectancies, assessed using 11 items.
						Implicit alcohol-related cognitions – Assessed using word completion task where words could be alcohol-related (e.g. *“bee…*; *spi…”*).
						Alcohol selection without purchasing - Hypothetical choice of beverage (from five alcoholic or ten non-alcoholic options).
Zwarun et al. (2006) [57]	USA	215 undergraduates; age not reported; 81 % female	Not reported	20 min of television programme (*“The Real World”* – a reality show) with beer advertisements embedded	20 min of same television programme with non-alcohol advertisements embedded	Explicit alcohol-related cognitions – Expectancies about the social benefits and physical effects of alcohol consumption (e.g. *“drinking makes me feel more confident”*, *“drinking alcohol relaxes me”*).

### Risk of bias

**Fig. 2:**
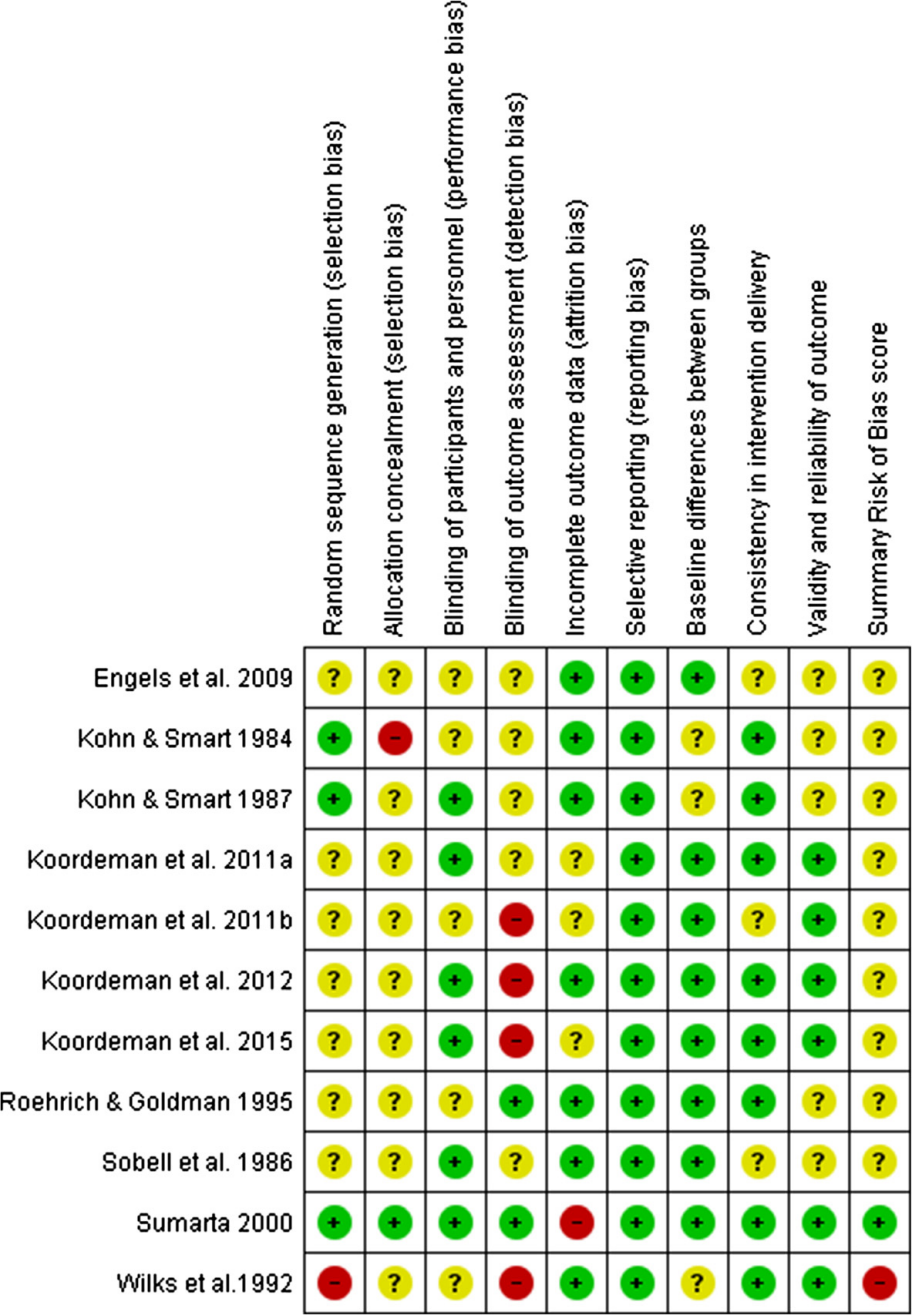
Risk of bias summary table. Review authors’ judgements about each risk of bias item for each included study assessing the primary outcome of alcohol consumption. Key: + indicates low risk; ? indicates unclear risk; − indicates high risk

Risk of bias assessments are summarised in Fig. 2. Detailed risk of bias tables are provided in Additional file 4. Study-level risk of bias was ‘unclear’ in nine of eleven studies that measured the primary outcome of alcohol consumption due to partial or unclear reporting of methods and procedures. One study [34] was judged to be at overall ‘low’ risk of bias (i.e. low risk of bias in all key domains) and the other [43] was judged to be at overall ‘high’ risk of bias due to concern about procedures applied to generate the allocation sequence.

At the domain-level, most studies reported insufficient information regarding randomisation procedures. Procedures for blinding participants were reported in six studies. Six of these studies [34, 37, 38, 40, 41, 43] reported successful blinding, indicated by responses to post-experiment questions regarding awareness of the study purpose, and the other [36] reported a blinding procedure but not whether this succeeded. Regarding blinding of outcome assessors, four of six studies with sufficient information were judged at ‘high’ risk of bias in this domain on the basis that measurement was conducted by assessors who were likely to have had knowledge of both the study hypotheses and participant assignments to conditions [39–41, 43, 44]. Eight studies [34, 35, 38–43] compared baseline differences between groups on demographic characteristics and typical alcoholic beverage consumption, with all but one finding no differences. The exception [35] found baseline differences in typical alcohol consumption and adjusted for these accordingly. Six studies reported information concerning the validity of the outcome measure [34, 38–41, 44]. All found that self-reported typical alcohol consumption was strongly associated with scores on the outcome measure, indicating that these scores were valid proxies of typical alcohol consumption.

### Effects on alcohol consumption

All eleven studies assessing the primary outcome of alcohol consumption were conducted using students, with ages ranging between 16 and 45 years. All studies received approval from institutional review boards, and all participants were above the legal drinking age in the country the study was conducted (16 being the legal drinking age in the Netherlands). Quantities of alcohol consumed were measured as millilitres of alcoholic beverage consumed, number of alcoholic drinks consumed (e.g. bottles of beer or glasses of wine), or both. When two measures of alcohol consumption were reported (e.g. number of bottles consumed *and* amount consumed in millilitres), we retained data for the more granular unit of measurement (e.g. amount consumed in millilitres). Two studies [34, 42] assessed consumption of non-alcoholic beer that participants had been led to believe contained alcohol.

#### Alcohol advertisements

**Fig. 3:**
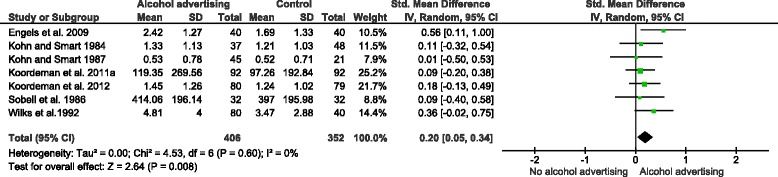
The effect of alcohol advertisements vs. control group interventions on alcohol consumption

Seven studies, with 758 participants, investigated the effect of exposure to alcohol advertisements on alcoholic beverage consumption [35–38, 40, 43, 44]. A random effects meta-analysis of data from these studies found a summary effect size (SMD) of 0.20 (95 % C.I. = 0.05–0.34), indicating that participants who viewed alcohol advertisements consumed more alcohol than those who did not, the size of this effect being small (Fig. 3).

For illustrative purposes, a summary effect size of 0.20 is equivalent to an increase of 1.57 (95 % C.I. = 0.39–2.67) alcohol units consumed by the average male drinker (around half a pint of lager at ABV 5.2 %), or an increase of 0.99 (95 % C.I. = 0.25–1.69) units consumed by the average female drinker (around half a 175 ml glass of wine at ABV 12 %), on the heaviest drinking day of the week.^1^

No statistical heterogeneity was observed (see Fig. 3), indicating that the direction and size of this effect was consistent between studies. Results were insensitive to the exclusion of data from one study [44] judged at high risk of bias (SMD = 0.17, 95 % C.I. = 0.01–0.32), and to the exclusion of two studies [36, 44] for which we had imputed standard deviations (SMD = 0.18, 95 % C. I. = 0.01–0.34). Results of random effects and fixed effects meta-analyses were similar. The number of participants included in this meta-analysis was lower than the threshold optimal information size (OIS) (i.e. the total number of participants generated by a conventional sample size calculation for a single adequately powered randomised controlled trial) when SMD = 0.20 (OIS = 788 at SMD = 0.20, α = 0.05, Power(1-β) = 0.8). Although visual inspection of the corresponding funnel plot did not indicate asymmetry, presence of publication bias cannot be ruled out.

**Fig. 4:**
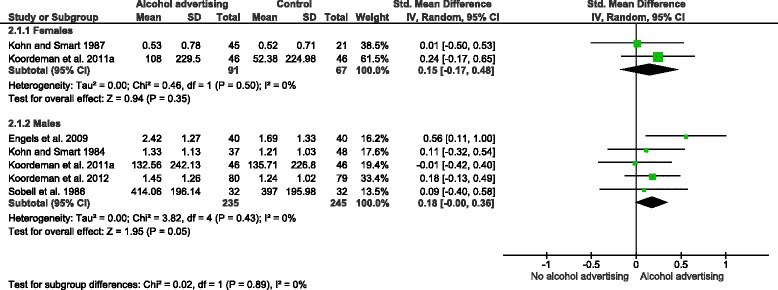
Sub-group analysis by gender: alcohol advertisements vs. control group interventions on alcohol consumption

Differences between male and female participants in the effect of exposure to alcohol advertising on consumption were investigated in a planned subgroup analysis. No differences between males and females were found (Fig. 4; *p* = 0.89), but this analysis was underpowered. Additionally, two studies reported on whether past week alcohol consumption moderated observed within-study effects. One of these studies [38] found that heavier past-week drinkers (>7 alcoholic beverages in past week) consumed more alcohol than lighter drinkers (≤7 drinks) following exposure to alcohol advertisements, whilst another [40] reported no differences in consumption between heavier and lighter drinkers.

#### Alcohol portrayals in television programmes or films

**Fig. 5:**
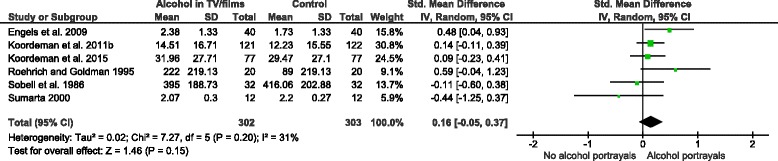
The effect of alcohol portrayals in television programmes or films vs. control group interventions on alcohol consumption

**Fig. 6:**
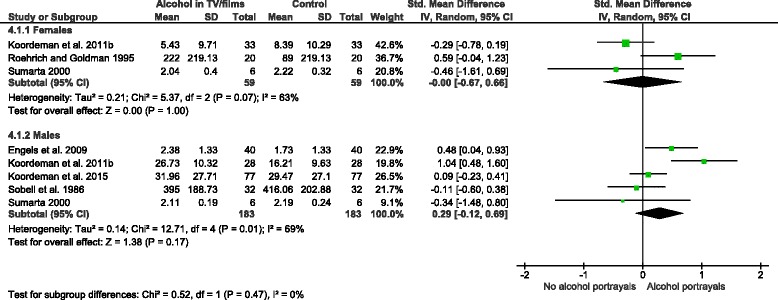
Subgroup analysis by gender: alcohol portrayals in television programmes or films

Six studies, with 605 participants, investigated the effect of exposure to alcohol portrayals in television programmes or films on immediate alcoholic beverage consumption [34, 35, 39, 41–43]. A random effects meta-analysis of these studies (Fig. 5) did not find any difference in quantities of alcoholic beverages consumed between exposed and unexposed participants (SMD = 0.16, 95 % C. I. = −0.05–0.37). The result was insensitive to the exclusion of outcome data from one study [42] that incorporated imputed standard deviations. Results of random and fixed effects meta-analyses were similar. The total number of participants was lower than the threshold optimal information size of 1,230. Visual inspection of the corresponding funnel plot did not indicate asymmetry, but again publication bias cannot be ruled out. A subgroup analysis found no difference in amounts consumed between males and females (Fig. 6; *p* = 0.47), but this analysis was again underpowered. One study reported whether self-control, a marker of executive function, moderated observed effects [41]. This study found no differences in consumption between high and low self-control participants in the alcohol portrayal condition.

### Effects on alcohol-related cognitions and alcohol selection

Fourteen included studies assessed the effects of exposure to alcohol marketing on at least one of the secondary outcomes of interest (Table 2). Explicit alcohol-related cognitions (explicit attitudes, outcome expectancies, or intentions to consume alcohol) were the most frequently measured construct (12 studies), whilst few included studies measured implicit alcohol-related cognitions (3 studies) or alcohol selection without purchase (4 studies). Three of these studies assessed multiple secondary outcomes [47, 50, 56]. Studies sampled college students (6), adolescent high school students (3), children between the ages of 8 and 12 (3), or adults from the general population (2). No included studies incorporated measures of alcohol purchasing.

#### Alcohol advertisements

**Fig. 7:**
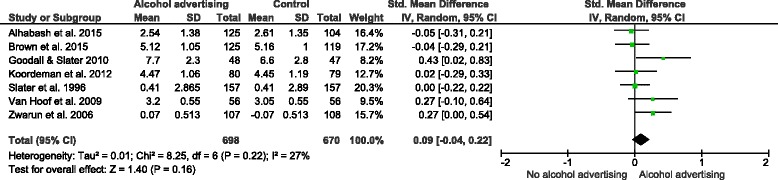
The effect of alcohol advertisements vs. control group interventions on explicit alcohol-related cognitions

Eight studies examined the impact of exposure to alcohol advertising on explicit alcohol-related cognitions [40, 45, 47, 49, 50, 55–57]. One study [49] reported finding an effect, but did not provide data in a form useable for meta-analysis. A random effects meta-analysis of data from the remaining seven studies (1,368 participants; Fig. 7) did not find a difference in positive explicit cognitions between exposed and unexposed participants (SMD = 0.09, 95 % C. I. = −0.04–0.22). The total sample size in this meta-analysis did not exceed the threshold optimal information size of 3,878.

**Fig. 8:**
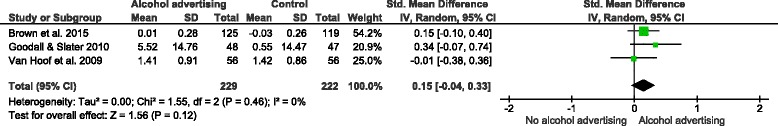
The effect of alcohol advertisements vs. control group interventions on implicit alcohol-related cognitions

Three studies assessed whether exposure to advertisements influenced implicit attitudes towards alcohol [47, 50, 56]. These studies each used different tasks to assess this construct. A random effects meta-analysis of data from these studies (451 participants, Fig. 8) did not find a difference between exposed and unexposed participants (SMD = 0.15, 95 % C. I. = −0.04–0.33). The total sample size in this meta-analysis did not exceed the threshold optimal information size of 1,398.

Three studies assessed whether exposure to advertisements influenced alcohol selection without purchasing [47, 53, 56]. One study [56] reported finding no effect on hypothetical selection of an alcoholic versus a non-alcoholic beverage amongst adolescents. One study [47] reported finding no effect on selection of a voucher for an alcohol-related versus non-alcohol related outlet among adults. One [53] tested whether viewing print-based alcohol advertisements influenced the amount of alcohol poured into mixed drinks, and also reported finding no effect. Data from these studies were not combined due to heterogeneity in the outcome measures used, and due to one study not providing useable data [56].

#### Alcohol portrayals in television programmes or films

**Fig. 9:**
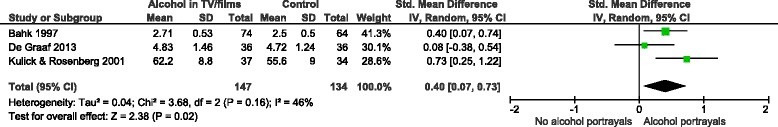
The effect of alcohol portrayals in television programmes or films vs. control group interventions on explicit alcohol-related cognitions

Four studies reported results concerning the impact of exposure to alcohol portrayals in television programmes or films on explicit alcohol-related cognitions [46, 48, 51, 52]. One study [51] reported not finding an effect, but did not provide useable data. A random effects meta-analysis of data from the remaining three studies (281 participants; Fig. 9) found that exposed participants reported more positively-valenced explicit cognitions than unexposed participants (SMD = 0.40, 95 % C. I. = 0.07–0.73). At the point estimate of this summary effect size (but not at the lower bound estimate of the 95 % confidence interval), the total sample size exceeded the threshold optimal information size of 200.

One study examined the effect of portrayals on alcohol selection without purchasing [54]. In this study, children (8–11 years old) were given a hypothetical choice of serving either whisky or water to photographs of adults. Participants who viewed a programme showing alcohol consumption were more likely to serve whisky than those who viewed the programme with no alcohol consumption and participants who viewed no television at all.

## Discussion

### Principal findings

The results of this systematic review suggest that exposure to alcohol advertisements may increase immediate consumption of alcoholic beverages by small amounts, equivalent to between 0.39 and 2.67 alcohol units for males and between 0.25 and 1.69 units for females. We did not find evidence that exposure to alcohol portrayals in television programmes or films had an effect on immediate alcoholic beverage consumption. No eligible studies investigating immediate effects of exposure to other forms of alcohol marketing on consumption were identified.

Confidence in summary estimates of these effects was diminished by three factors. First, our meta-analyses were typically underpowered. Second, the lower bound confidence interval for our estimate of the effect of advertisements on consumption was close to the line of no effect. Third, risk of bias remained unclear in the majority of source studies due to incomplete or ambiguous reporting of study design features, methods and procedures. Taken together, these factors leave open the possibility that the integration of results from further research could substantively change summary estimates of these effect sizes, and hence the principal findings of this review.

Our findings are, however, broadly consistent with those of previous systematic reviews on this topic, which report evidence for a positive association between exposure to alcohol marketing, of any type and over time, and quantities of alcoholic beverages consumed [6–8]. Our findings support the less emphatic claim that exposure to alcohol advertising, but not alcohol portrayals in television programmes or films, may increase quantities consumed immediately following exposure, by small amounts. This difference in findings might reflect the possibility that a series of discrete exposures, each of which individually causes a relatively small or no *immediate* increase in consumption, cumulate into higher levels of consumption over time among more exposed people. Alternatively, our findings may be specific to student populations, the only participants in the studies included in this review, so may not generalize to the general populations included in longitudinal studies. The distinction found in this review between the impact of alcohol advertising and that of media portrayals of alcohol use may be due to the media portrayals used not being genuine attempts at alcohol marketing at all, but rather incidental to the storylines of the television programmes and films presented and so not designed to elicit any behavioural response from the viewer. It was not made clear in primary studies whether media portrayals used were genuine examples of alcohol marketing.

Overall, the reviewed body of evidence for the immediate effects of alcohol marketing communications and media portrayals did not contribute much to elucidating differences in effects (moderation) by participant characteristics. First, included studies that measured alcohol consumption were invariably conducted among recruited samples of students. Second, a planned subgroup analysis did not identify any difference in the effect on consumption between males and females but was underpowered; while other planned subgroup analyses were precluded by lack of reporting of relevant outcome data in a useable form. Third, regarding variation by typical drinking habits, one study found that effects of alcohol advertising on alcohol consumption were larger in heavier drinkers, whilst another found no differences between lighter and heavier drinkers; and regarding variation by individual differences in executive function, one study reported no differences between participants high or low in self-control (the ability to resist impulses that conflict with long-term goals, and a marker of executive function [58]) in alcohol consumed after exposure to alcohol portrayals.

Findings were also equivocal concerning the effects of exposure to alcohol marketing communications on alcohol-related cognitions (proposed mediators of the effect on consumption). We found evidence that exposure to alcohol portrayals increased positively valenced explicit alcohol-related cognitions, but did not find evidence that exposure to alcohol advertisements influenced explicit or implicit alcohol-related cognitions. We did not identify any eligible studies that assessed the impact of exposure to alcohol portrayals on implicit alcohol-related cognitions. Viewing media portrayals of alcohol consumption in positive contexts and with positive consequences such as companionship, bonding, and celebration appears to increase general liking of alcohol and expectations that consumption will result in positive outcomes. However, this finding is based on data from only three studies. Additional limitations of meta-analyses for secondary outcomes included heterogeneous participants and specific outcome measures used in primary studies, and lack of statistical precision (especially for the two meta-analyses pertaining to alcohol advertisements). As such, current evidence from experimental studies neither undermines nor convincingly supports the proposal that exposure to alcohol marketing influences subsequent alcohol consumption by first inducing (and then, by repeated exposure, reaffirming) positive alcohol-related cognitions.

### Strengths and limitations

The strengths of this systematic review lie in its methodological rigour and its novel focus on experimental studies with objectively measured alcohol consumption as the primary outcome. However, scope to fully address the review’s primary objective was limited by the overall completeness and applicability of the evidence base. In particular, we identified an absence of published experimental research into the effects of alcohol marketing delivered using channels other than visual broadcast media and involving participants other than students. The generalizability of our findings beyond these channels and this specific population subgroup has therefore yet to be established. Scope to address the review’s secondary objective, to investigate factors that might explain observed heterogeneity in effects between studies, was severely hampered by the narrow coverage of possible moderating factors in included studies, coupled with the lack of reporting of outcome data in the disaggregated form needed for planned subgroup analyses. A broader limitation of the evidence base is that individual studies tended to measure general effects of alcohol marketing communications with little or no consideration of how the creative content of individual communications is tailored to influence individuals with specific psychosocial characteristics, or how differences in individual receptivity to marketing content might modify any effects.

### Implications for policy

Harmful alcohol consumption is responsible for 5.9 % of all deaths worldwide, accounts for 5.1 % of the global burden of injury and disease, and brings substantial social and economic costs to individuals and societies [59]. Restricting the marketing of alcoholic products, along with increasing price and reducing availability, is purported to be a cost-effective intervention to reduce consumption [60]. There is a lack of high quality evidence for or against the implementation of such interventions [61]. As such, alcohol marketing restrictions are not favoured by policy makers [62]. Whilst the evidence from experimental studies is currently limited, the results of this systematic review do, in our view, lend some qualified support to the public health case for restrictions, bans, or other policies that would reduce exposure to alcohol advertising on visual broadcast media to reduce alcohol consumption. Importantly, whilst the individual-level immediate effects found here may be small, such effects could, if sustained in response to overall reduced exposure over time, have a meaningful impact on consumption at the population level. Whilst the evidence synthesised in the current review concerns only the immediate effects of alcohol marketing and media exposure, the findings can be considered alongside those from systematic reviews of longitudinal studies, which imply that less exposure to alcohol marketing would lead to a later age of starting to drink alcohol and lower alcohol consumption in young people.

### Implications for research

There is a need for high quality experimental and quasi-experimental studies into the effects of alcohol marketing on alcohol consumption that focus on media channels other than broadcast media, particularly in populations other than undergraduate students, and powered for main effects and subgroup analyses. In recent years there has been considerable development in the subtlety and range of alcohol marketing communications [63–65]. Research into the effects of alcohol marketing has failed to keep pace with these developments. More work is required regarding how effects of alcohol marketing on consumption might be mediated by implicit processes, or modified by individual differences. Finally, we recommend more detailed reporting of future experimental studies of the acute effects of alcohol marketing exposure, so that risk of bias to their results can be comprehensively assessed.

## Conclusions

Current evidence from experimental studies suggests that exposure to alcohol advertisements may increase the quantities of alcoholic beverages that people immediately consume by small amounts. Current evidence does not support the claim that exposure to portrayals of alcohol use in films and television programmes increases immediate alcohol consumption. Exposure to alcohol portrayals, but not alcohol advertisements, may increase positive explicit alcohol-related cognitions. It remains to be established whether these findings generalize beyond students and to other marketing channels.

## Additional files

Additional file 1: Electronic searches, dates and yields. (DOCX 54 kb) Additional file 2: Characteristics of excluded studies. (DOCX 14 kb) Additional file 3: Characteristics of included studies. (DOCX 100 kb) Additional file 4: Risk of bias judgements. (DOCX 31 kb)

## Abbreviations

OIS: Optimal information size
SMD: Standardized mean difference

## Footnotes

1 Calculated by multiplying our summary effect size and 95 % confidence intervals by the standard deviation of mean alcohol unit consumption for males (mean = 8.39 units ± 7.85) and females (mean = 5.33 units ± 4.96) on the heaviest drinking day of the week amongst alcohol users over the age of 18 in the United Kingdom. Consumption data taken from the National Diet and Nutrition Survey, 2014 [71].
