# Supplementary material for: Immediate effects of alcohol marketing communications and media portrayals on consumption and cognition: a systematic review and meta-analysis of experimental studies
Source: BMC Public Health. 2016 Jun 9;16:465. doi: 10.1186/s12889-016-3116-8 (PMC4899920; doi:10.1186/s12889-016-3116-8)
Supplement: Additional file 1: — Electronic searches, dates and yields. (DOCX 54 kb) [file 12889_2016_3116_MOESM1_ESM.docx]

Additional file 1.

*Electronic searches, dates and yields*

**MEDLINE (EbscoHost)**

Date of initial search: 22/11/2013

Search yield: 5,329 records (3,017 unique)

Date of updated search: 17/09/2015

Search yield: 1,399 records

1. experimental design/

2. experimental psychology/

3. random allocation/

4. randomized controlled trial/

5. experiment$.ti,ab.

6. effect$.ti,ab.

7. (random$ adj1 sampl$).ti,ab.

8. (random$ adj1 assign$).ti,ab.

9. (independent adj1 variable$).ti,ab.

10. (dependent adj1 variable$).ti,ab.

11. (control adj1 group$).ti,ab.

12. (control adj1 condition$).ti,ab.

13. (between adj1 subject$).ti,ab.

14. (between adj1 participant$).ti,ab.

15. (within adj1 subject$).ti,ab.

16. (within adj1 participant$).ti,ab.

17. comparative study.pt.

18. or/1-17

19. animal experimentation/

20. exp animals/ not humans.sh.

21. or/19-20

22. 18 not 21

23. advertising as topic/

24. mass media/

25. social media/

26. social marketing/

27. communication/

28. cues/

29. marketing.ti,ab.

30. sponsorship.ti,ab.

31. advert$.ti,ab.

32. media.ti,ab.

33. communicat$.ti,ab.

34. commercial$1.ti,ab.

35. (product adj1 placement).ti,ab.

36. point of purchase.ti,ab.

37. point of sale.ti,ab.

38. audio-visual.ti,ab.

39. audiovisual.ti,ab.

40. print.ti,ab.

41. television.ti,sh,ab,kw.

42. tv.ti,ab.

43. radio.ti,sh,ab,kw.

44. cinema$1.ti,ab.

45. movie$.ti,ab.

46. (motion adj1 picture$1).ti,ab.

47. newspaper$1.ti,sh,ab,kw.

48. magazine$1.ti,ab,kw.

49. poster$1.ti,ab.

50. billboard$1.ti,ab.

51. flyer$1.ti,ab.

52. (social adj1 network$).ti,ab.

53. (social adj1 media).ti,ab.

54. (new adj1 media).ti,ab.

55. internet.ti,sh,ab,kw.

56. online.ti,ab,kw.

57. website$1.ti,ab,kw.

58. (mobile adj1 phone$1).ti,ab.

59. (cell adj1 phone$1).ti,ab.

60. or/23-59

61. exp alcoholic beverages/

62. exp drinking behavior/

63. exp alcohol drinking/

64. alcohol$.ti,ab.

65. (binge adj1 drinking).ti,ab.

66. beer$1.ti,ab,kw.

67. lager$1.ti,ab.

68. wine$1.ti,ab,kw.

69. cider$1.ti,ab.

70. alcopop$1.ti,ab.

71. spirit$1.ti,ab.

72. liq?r$1.ti,ab,kw.

73. whisk?y$1.ti,ab.

74. vodka$1.ti,ab.

75. brandy.ti,ab.

76. gin$1.ti,ab.

77. rum$1.ti,ab.

78. tequila$1.ti,ab,kw.

79. schnapps.ti,ab.

80. cocktail$1.ti,ab.

81. or/61-80

82. 22 and 60 and 81

**Embase (Ovid SP)**

Date of initial search: 15/11/2013

Search yield: 3,048 records (2,144 unique)

Date of updated search: 17/09/2015

Search yield: 976 records

1. human experiment/

2. experimental design/

3. random sample/

4. randomization/

5. randomized controlled trial/

6. independent variable/

7. dependent variable/

8. control group/

9. experiment$.ti,ab.

10. (random$ adj1 sampl$).ti,ab.

11. (random$ adj1 alloca$).ti,ab.

12. (random$ adj1 assign$).ti,ab.

13. (independent adj1 variable$).ti,ab.

14. (dependent adj1 variable$).ti,ab.

15. (control adj1 group$).ti,ab.

16. (control adj 1 condition$).ti,ab.

17. (between adj1 subject$).ti,ab,kw.

18. (between adj1 participant$).ti,ab,kw.

19. (within adj1 subject$).ti,ab,kw.

20. (within adj1 participant$).ti,ab,kw.

21. or/1-20

22. animal/

23. human/ and animal/

24. 22 not 23

25. 21 not 24

26. advertizing/

27. exp mass communication/

28. marketing/

29. social marketing/

30. television viewing/

31. association/

32. marketing.ti,ab.

33. sponsorship.ti,ab.

34. advert$.ti,ab.

35. communicat$.ti,ab.

36. commercial$1.ti,ab,kw.

37. cue$.ti,ab.

38. (product adj1 placement).ti,ab.

39. point of sale.ti,ab.

40. point of purchase.ti,ab.

41. audio-visual.ti,ab.

42. audiovisual.ti,ab.

43. print.ti,ab.

44. television.ti,ab,kw.

45. tv.ti,ab.

46. radio.ti,ab,kw.

47. cinema$1.ti,ab.

48. movie$.ti,ab.

49. (motion adj1 picture$1).ti,ab.

50. newspaper$1.ti,ab,kw.

51. magazine$1.ti,ab,kw.

52. poster$1.ti,ab.

53. billboard$1.ti,ab.

54. flyer$1.ti,ab.

55. (social adj1 network$).ti,ab.

56. (social adj1 media).ti,ab.

57. (new adj1 media).ti,ab.

58. internet.ti,ab,kw.

59. online.ti,ab,kw.

60. website$1.ti,ab,kw.

61. (mobile adj1 phone$1).ti,ab.

62. (cell$ adj1 phone$1).ti,ab.

63. or/26-62

64. exp alcoholic beverage/

65. drinking behavior/

66. binge drinking/

67. alcohol consumption/

68. alcohol$.ti,ab.

69. (binge adj1 drinking).ti,ab.

70. beer$1.ti,ab,kw.

71. lager$1.ti,ab.

72. wine$1.ti,ab,kw.

73. cider$1.ti,ab.

74. alcopop$1.ti,kw.

75. spirit$1.ti,ab.

76. liqu?r$1.ti,ab,kw.

77. whisk?y$1.ti,ab.

78. vodka$1.ti,ab.

79. brandy.ti,ab.

80. gin$1.ti,ab.

81. rum$1.ti,ab.

82. tequila$1.ti,ab,kw.

83. schnapps.ti,ab.

84. cocktail$1.ti,ab.

85. or/64-84

86. 25 and 63 and 85

**PsycInfo (Ovid SP)**

Date of initial search: 29/11/2013

Search yield: 2,917 records (2,314 unique)

Date of updated search: 17/09/2015

Search yield: 917 records

1. experimental design/

2. experimental psychology/

3. random sampling/

4. independent variables/

5. dependent variables/

6. experiment controls/

7. experiment$.ti,ab.

8. effect$.ti,ab.

9. (random$ adj1 sampl$).ti,ab.

10. (random$ adj1 alloca$).ti,ab.

11. (random$ adj1 assign$).ti,ab.

12. (independent adj1 variable$).ti,ab.

13. (dependent adj1 variable$).ti,ab.

14. (control adj1 group$).ti,ab.

15. (control adj1 condition$).ti,ab.

16. (between adj1 subject$).ti,ab.

17. (between adj1 participant$).ti,ab.

18. (within adj1 subject$).ti,ab.

19. (within adj1 participant$).ti,ab.

20. or/1-19

21. exp animals/

22. 20 not 21

23. advertising/

24. exp communications media/

25. exp marketing/

26. exp electronic communication/

27. television viewing/

28. websites/

29. internet/

30. cues/

31. marketing.ti,ab.

32. sponsorship.ti,ab.

33. advert$.ti,ab.

34. media.ti,ab.

35. communicat$.ti,ab.

36. commercial$1.ti,ab.

37. (product adj1 placement).ti,ab.

38. point of purchase.ti,ab.

39. point of sale.ti,ab.

40. audio-visual.ti,ab.

41. audiovisual.ti,ab.

42. print.ti,ab.

43. television.ti,ab.

44. tv.ti,ab.

45. radio.ti,ab.

46. cinema$1.ti,ab.

47. movie$.ti,ab.

48. (motion adj1 picture$1).ti,ab.

49. newspaper$1.ti,ab.

50. magazine$1.ti,ab.

51. poster$1.ti,ab.

52. billboard$1.ti,ab.

53. flyer$1.ti,ab.

54. (social adj1 network$).ti,ab.

55. (social adj1 media).ti,ab.

56. (new adj1 media).ti,ab.

57. internet.ti,ab.

58. online.ti,ab.

59. website$1.ti,ab.

60. (mobile adj1 phone$1).ti,ab.

61. (cell$ adj1 phone$1).ti,ab.

62. or/23-61

63. exp alcoholic beverages/

64. exp alcohol drinking patterns/

65. Binge Drinking/

66. alcohol$.ti,ab.

67. (binge adj1 drinking).ti,ab.

68. beer$1.ti,ab.

69. lager$1.ti,ab.

70. wine$1.ti,ab.

71. cider$1.ti,ab.

72. alcopop$1.ti,ab.

73. spirit$1.ti,ab.

74. liq?r$1.ti,ab.

75. whisk?y$1.ti,ab.

76. vodka$1.ti,ab.

77. brandy.ti,ab.

78. gin$1.ti,ab.

79. rum$1.ti,ab.

80. tequila$1.ti,ab.

81. schnapps.ti,ab.

82. cocktail$1.ti,ab.

83. or/63-82

84. 22 and 62 and 83

**ASSIA (ProQuest)**

Date of initial search: 27/11/2013

Search yield: 625 records (306 unique)

Date of updated search: 17/09/2015

Search yield: 163 records

(SU.EXACT("Experiments") OR SU.EXACT("Experimental psychology") OR SU.EXACT("Randomization") OR SU.EXACT("Random sampling") OR SU.EXACT("Randomized controlled trials") OR SU.EXACT("Independent variables") OR SU.EXACT("Control groups") OR RTYPE("comparative study") OR AB,TI(experiment*) OR AB,TI(effect*) OR AB,TI(random* NEAR/1 sampl*) OR AB,TI(random* NEAR/1 assign*) OR AB,TI(random* NEAR/1 allocat*) OR AB,TI(independent* NEAR/1 variable*) OR AB,TI(dependent* NEAR/1 variable*) OR AB,TI(control NEAR/1 group*) OR AB,TI(control NEAR/1 condition*) OR AB,TI(between* NEAR/1 subject*) OR AB,TI(between* NEAR/1 participant*) OR AB,TI(within* NEAR/1 subject*) OR AB,TI(within* NEAR/1 participant*)) AND (SU.EXACT.EXPLODE("Advertising" OR "Billboards") OR SU.EXACT.EXPLODE("Advertisements" OR "Films" OR "Mass media" OR "Newspapers" OR "Posters" OR "Press" OR "Radio" OR "Television") OR SU.EXACT("Marketing") OR SU.EXACT("Communication") OR SU.EXACT("Sponsorship") OR SU.EXACT("Television viewing") OR SU.EXACT("Television programmes") OR SU.EXACT("Internet") OR SU.EXACT.EXPLODE("Telecommunications") OR SU.EXACT("Cinemas") OR SU.EXACT("Mobile phones") OR SU.EXACT("Cue exposure") OR SU.EXACT.EXPLODE("Alcohol related cues" OR "Cues" OR "Precuing" OR "Social cues" OR "Verbal cues" OR "Visual cues") OR AB,TI(marketing) OR AB,TI(sponsorship) OR AB,TI(Advert*) OR AB,TI(communicat*) OR AB,TI(commercial*1) OR AB,TI(cue*) OR AB,TI(product NEAR/1 placement) OR AB,TI("point of purchase") OR AB,TI("point of sale") OR AB,TI("audio-visual") OR AB,TI("audio visual") OR AB,TI(print) OR AB,TI("television") OR AB,TI(tv) OR AB,TI(movie*) OR AB,TI(motion NEAR/1 picture) OR AB,TI(newspaper*1) OR AB,TI(magazine*1) OR AB,TI(poster*1) OR AB,TI(billboard*1) OR AB,TI(flyer*1) OR AB,TI(social NEAR/1 network*) OR AB,TI(social NEAR/1 media) OR AB,TI(new NEAR/1 media) OR AB,TI(internet) OR AB,TI(online) OR AB,TI(website*1) OR AB,TI(cell* NEAR/1 phone)) AND (SU.EXACT.EXPLODE("Alcohol consumption" OR "Bars" OR "Binge drinking" OR "Social drinking") OR SU.EXACT.EXPLODE("Alcoholic beverages" OR "Alcoholic soft drinks" OR "Beer" OR "Spirits" OR "Wine") OR SU.EXACT("Alcohol intoxication") OR AB,TI(alcohol*) OR AB,TI(drinking NEAR/1 behavio?r*) OR AB,TI(alcohol*) OR AB,TI(beer*1) OR AB,TI(lager*1) OR AB,TI(wine*1) OR AB,TI(cider*1) OR AB,TI(alcopop*1) OR AB,TI(spirits*1) OR AB,TI(liqu?r*1) OR AB,TI(whisk?y*1) OR AB,TI(vodka*1) OR AB,TI(brandy) OR AB,TI(gin*1) OR AB,TI(rum*1) OR AB,TI(tequila*1) OR AB,TI(schnapps) OR AB,TI(cocktail*1))

**Web of Science (Thompson Reuters)**

- Science Citation Index Expanded
- Social Sciences Citation Index
- Conference Proceedings Citation Index - Science
- Conference Proceedings Citation Index - Social Sciences & Humanities

Date of initial search: 16/11/2013

Search yield: 4,884 records (3,422 unique)

Date of updated search: 17/09/2015

Search yield: 1,671 records

(((TS=(experiment* OR effect* OR random*) OR TS=(independent NEAR/1 variable*) OR TS=(dependent NEAR/1 variable*) OR TS=(control NEAR/1 group*) OR TS=(control NEAR/1 condition*) OR TS=(between NEAR/1 subject*) OR TS=(between NEAR/1 participant*) OR TS=(within NEAR/1 subject*) OR TS=(within NEAR/1 participant*)) AND (TS=(marketing OR sponsorship* OR advert* OR media OR communicat* OR commercial* OR audio-visual OR audiovisual OR print OR television OR tv OR radio OR cinema* OR movie* OR newspaper* OR magazine* OR poster* OR billboard* OR flyer* OR internet* OR online OR website* OR cue*) OR TS=(product NEAR/1 placement) OR TS=(point NEAR/2 purchase) OR TS=(point NEAR/2 sale) OR TS=(motion NEAR/1 picture*) OR TS=(social NEAR/1 network*) OR TS=(social NEAR/1 media) OR TS=(new NEAR/1 media) OR TS=(mobile NEAR/1 phone*) OR TS=(cell* NEAR/1 phone*)) AND (TS=(alcohol* OR beer* OR lager* OR wine* OR cider* OR alcopop* OR spirit* OR liq*r* OR whisk*y* OR vodka* OR brandy OR gin* OR rum* OR tequila* OR schnapps OR cocktail*) OR TS=(drinking NEAR/1 behavio*r*) OR TS=(drinking NEAR/1 pattern*) OR TS=(binge NEAR/1 drinking)) AND (WC=(Behavioral Sciences OR Business OR Communication OR Film, Radio, Television OR Health Care Sciences & Services OR Health Policy & Services OR Management OR Medicine, Research & Experimental OR Multidisciplinary Sciences OR Nutrition & Dietetics OR Physiology OR Primary Health Care OR Psychology OR Psychology, Applied OR Psychology, Biological OR Psychology, Clinical OR Psychology, Experimental OR Psychology, Multidisciplinary OR Psychology, Social OR Public, Environmental & Occupational Health OR Social Issues OR Social Sciences, Interdisciplinary OR Substance Abuse))) NOT (TS=(animal model* OR animal* OR animal experiment* OR animal disease model* OR laboratory animal*))) AND Language=(English) AND Document Types=(Article OR Abstract of Published Item OR Book OR Book Chapter OR Proceedings Paper OR Review)

*Databases=SCI-EXPANDED, SSCI, CPCI-S, CPCI-SSH, CCR-EXPANDED, IC Timespan=All years*

**Econlit (EBSCOhost)**

Date of initial search: 15/11/2013

Search yield: 719 records (687 unique)

Date of updated search: 17/09/2015

Search yield: 121 records

((TI (marketing OR sponsor* OR advert* OR media OR communicat* OR commercial* OR audio-visual OR audiovisual OR print OR television OR tv OR radio OR cinema* OR movie* OR newspaper* OR magazine* OR poster* OR billboard* OR flyer* OR internet* OR online OR website* OR cue* OR "product placement" OR "point of purchase" OR "point of sale" OR "motion picture* " OR "social network*" OR "social media" OR "new media" OR "mobile phone*" OR "cell* phone*")) OR (AB (marketing OR sponsor* OR advert* OR media OR communicat* OR commercial* OR audio-visual OR audiovisual OR print OR television OR tv OR radio OR cinema* OR movie* OR newspaper* OR magazine* OR poster* OR billboard* OR flyer* OR internet* OR online OR website* OR cue* OR "product placement" OR "point of purchase" OR "point of sale" OR "motion picture* " OR "social network*" OR "social media" OR "new media" OR "mobile phone*" OR "cell* phone*"))) AND ((TI (alcohol* OR drinking OR drink OR beer* OR lager* OR wine* OR cider* OR alcopop* OR spirit* OR liq*r* OR whisk*y* OR vodka* OR brandy OR gin* OR rum* OR tequila* OR schnapps OR cocktail*)) OR (AB (alcohol* OR drinking OR drink OR beer* OR lager* OR wine* OR cider* OR alcopop* OR spirit* OR liq*r* OR whisk*y* OR vodka* OR brandy OR gin* OR rum* OR tequila* OR schnapps OR cocktail*)))
