# Supplementary material for: Immediate effects of alcohol marketing communications and media portrayals on consumption and cognition: a systematic review and meta-analysis of experimental studies
Source: BMC Public Health. 2016 Jun 9;16:465. doi: 10.1186/s12889-016-3116-8 (PMC4899920; doi:10.1186/s12889-016-3116-8)
Supplement: Additional file 2: — Characteristics of excluded studies. (DOCX 14 kb) [file 12889_2016_3116_MOESM2_ESM.docx]

Additional file 2

*Characteristics of excluded studies*

| **Study** | **Reason for Exclusion** |
| --- | --- |
| Atkin & Block (1984) | **Not an eligible study design:** Judged unlikely to be a randomised experiment (inadequate reporting of design) |
| Austin & Johnson (1997) | **No eligible interventions:** Assesses effects of media literacy training (using marketing), not the effects of pro alcohol marketing per se. |
| Bohannon et al. (1993) | **No eligible interventions:** Assesses the effects of alcohol warnings only, not pro alcohol marketing. |
| Christie et al. (2001) | **Not an eligible study design:** No randomisation. |
| Garretson & Burton (1998) | **Not an eligible study design:** No randomisation. |
| Kohn, Smart & Ogborne (1984) | **Not an eligible study design:** Field experiment (not laboratory based). |
| MacKinnon & Lapin (1998) | **Not an eligible study design:** No randomisation. |
| MacKinnon (1993) | **Not an eligible study design:** No randomisation. |
| Russell & Russell (2008) | **Not an eligible study design:** No randomisation. |
| Selvanathan (1995) | **Not an eligible study design:** No randomisation. |
| Slater et al. (1997) | **Not an eligible study design:** No randomisation. |
| Slater (1996) | **No measurement (assessment) of eligible primary or secondary outcomes:** Perceptions of advertisements. |
| Slater et al. (1996) | **No measurement (assessment) of eligible primary or secondary outcomes:** Cognitive resistance (counterarguments) against advertisement. |
| Wyllie et al. (1998) | **Not an eligible study design:** Telephone/survey design (not laboratory based). |
