# Supplementary material for: Immediate effects of alcohol marketing communications and media portrayals on consumption and cognition: a systematic review and meta-analysis of experimental studies
Source: BMC Public Health. 2016 Jun 9;16:465. doi: 10.1186/s12889-016-3116-8 (PMC4899920; doi:10.1186/s12889-016-3116-8)
Supplement: Additional file 3: — Characteristics of included studies. (DOCX 100 kb) [file 12889_2016_3116_MOESM3_ESM.docx]

Additional file 3

*Characteristics of included studies*

| **Alhabash, McAlister, Quilliam, Richards, & Lou (2015)** | |
| --- | --- |
| **Methods** | Randomised, online experiment (between-subjects design) |
| **Participants** | **Geographical region:** USA, Midwest  **Sampling frame:** University  **Sampling method:** Opportunity sample (Course credit)  **Number of participants randomised:** 413  **Number (%) of enrolled participants completing the study:** 379 (91.8%)  **Study completers - Mean age (SD):** 20.58 (1.52)  **Study completers - Sex (Female %):** 57.1  **Ethnicity:** “mostly White/Caucasian (77.2%)”  **Socio-economic status:** Not reported  **Education:** A-Level (Level 3) - Inferred from undergraduate student status  **Baseline alcohol consumption:** Not reported  **Executive function:** Not reported  **Inclusion criteria:** Not reported  **Exclusion criteria:** Participants whose data points were within 2 standard deviations of the mean on all variables |
| **Interventions** | **Intervention 1 –**  **Setting:** Online, via Qualtrics viewing platform  **Valence:** Pro  **Medium:** Social media - Facebook  **Title:** N/A  **Total duration of presentation:** “Participants took, on average, 1 hour to finish the study.”  **Number of portrayals:** 6  **Duration of portrayals:** “participants were instructed to take as much time as they like to view each page”  **Brands presented:** "a happy hour at local restaurants"  **Type of alcohol presented:** Not reported  **Editing details:** "Stimuli were specifically constructed for this experiment."  **Intervention 2 –**  **Setting:** Online, via Qualtrics viewing platform  **Valence:** Anti  **Medium:** Social media - Facebook  **Title:** N/A  **Total duration of presentation:** “Participants took, on average, 1 hour to finish the study.”  **Number of portrayals:** 6  **Duration of portrayals:** “participants were instructed to take as much time as they like to view each page”  **Brands presented:** "an anti-binge-drinking PSA"  **Type of alcohol presented:** Not reported  **Editing details:** "Stimuli were specifically constructed for this experiment."  **Control -**  **Setting:** Online, via Qualtrics viewing platform  **Valence:** Neutral  **Medium:** Social media - Facebook  **Title:** N/A  **Total duration of presentation:** “Participants took, on average, 1 hour to finish the study.”  **Number of portrayals:** 6  **Duration of portrayals:** “participants were instructed to take as much time as they like to view each page”  **Brands presented:** "a local financial institution"  **Type of alcohol presented:** Not reported  **Editing details:** "Stimuli were specifically constructed for this experiment."  **Additional details -**  **Intervention arms:** *Intervention 1* – Alcohol advertising, *Control* – Neutral advertising  **Comparisons analysed:** Conditions analysed separately (included other within-condition variables not relevant to this review).  **Concurrent intervention components:** None |
| **Outcomes** | **Outcomes reported in study:** Explicit alcohol-related cognitions** - Intentions to consume alcohol (**Task:** Questionnaire, **Unit of measurement:** Four items with 7-point response scale)  **Timing of outcome measurement:** Following each screenshot  **Additional outcomes:** Attitudes towards the ad; intentions to share ad  ** Primary outcome*  *** Secondary Outcome* |
| **Notes** |  |

| **Bahk (1997)** | |
| --- | --- |
| **Methods** | Randomised, laboratory-based experiment (between-subjects design) |
| **Participants** | **Geographical region:** USA, Midwest  **Sampling frame:** University  **Sampling method:** Opportunity sample (Course credit)  **Number of participants randomised:** 224  **Number (%) of enrolled participants completing the study:** 211 (94.2%) 73 Intervention 1, 74 intervention 2, 64 control  **Study completers - Mean age (SD):** 19.81 (1.53)  **Study completers - Sex (Female %):** 64.29  **Ethnicity:** Not reported  **Socio-economic status:** Not reported  **Education:** A-Level (Level 3) - Inferred from undergraduate student status  **Baseline alcohol consumption:** Not reported  **Executive function:** Not reported  **Inclusion criteria:** Not reported  **Exclusion criteria:** Prior exposure to the film "A Star Is Born" |
| **Interventions** | **Intervention 1 –**  **Setting:** University "viewing room" equipped with TV and chairs  **Valence:** Pro  **Medium:** Film  **Title:** “A Star Is Born”  **Total duration of presentation:** 32 minutes  **Number of portrayals:** Not reported  **Duration of portrayals:** 5 minutes  **Brands presented:** Not reported  **Type of alcohol presented:** Not reported  **Editing details:** Film edited to remove negative consequences of alcohol  **Intervention 2 –**  **Setting:** University "viewing room" equipped with TV and chairs  **Valence:** Anti  **Medium:** Film  **Title:** “A Star Is Born”  **Total duration of presentation:** 36 minutes  **Number of portrayals:** Not reported  **Duration of portrayals:** 4 minutes  **Brands presented:** Not reported  **Type of alcohol presented:** Not reported  **Editing details:** No editing  **Control -**  **Setting:** University "viewing room" equipped with TV and chairs  **Valence:** Neutral  **Medium:** Film  **Title:** “A Star Is Born”  **Total duration of presentation:** 27 minutes  **Number of portrayals:** N/A  **Duration of portrayals:** N/A  **Brands presented:** Not reported  **Type of alcohol presented:** N/A  **Editing details:** Film edited to remove both positive and negative consequences of alcohol consumption  **Additional details -**  **Intervention arms:** *Intervention 1* - Edited film clip showing positive consequences of alcohol consumption only; *Intervention 2* - Edited film clip showing positive and negative consequences of alcohol consumption; *Control* - Edited film clip showing neither the positive or negative consequences of alcohol consumption  **Comparisons analysed:** Intervention 1 v Control; Intervention 2 v Control; Intervention 1 v Intervention 2  **Concurrent intervention components:** None |
| **Outcomes** | **Outcomes reported in study:** Explicit alcohol-related cognitions** - Attitudes (**Task:** Questionnaire, **Unit of measurement:** Fifteen 5-point scale items)  **Timing of outcome measurement:** Immediately after experimental manipulation  **Additional outcomes:** Not reported  ** Primary outcome*  *** Secondary Outcome* |
| **Notes** |  |

| **Brown, Stautz, Hollands, Winpenny, & Marteau (2015)** | |
| --- | --- |
| **Methods** | Randomised, online experiment (between-subjects design) |
| **Participants** | **Geographical region:** UK  **Sampling frame:** Adults  **Sampling method:** Research agency online survey panel  **Number of participants randomised:** 540  **Number (%) of enrolled participants completing the study:** 373 (69.1%)  **Study completers - Mean age (SD):** 28.03 (5.64)  **Study completers - Sex (Female %):** 59.5  **Ethnicity:** 315 White British, 7 White Irish, 21 White other, 2 Mixed White and Black African, 5 Mixed White and Asian, 2 Mixed other, 4 Indian, 4 Pakistani, 3 Other Asian/Asian British, 4 Black/Black British, 6 Chinese  **Socio-economic status:** Mean Index of Multiple Deprivation=21.08 (SD=15.07)  **Education:** Highest educational qualification: 194 degree, 92 A-levels, 54 5+GCSEs, 20<GCSEs, 7 no qualifications  **Baseline alcohol consumption:** Mean 16.02 (SD=15.07) units past week  **Executive function:** Mean Stop Signal Reaction Time = 272.57 (SD=80.27)  **Inclusion criteria:** Not reported  **Exclusion criteria:** Participants whose data points were outside 3 standard deviations of the mean on baseline alcohol consumption |
| **Interventions** | **Intervention 1 –**  **Setting:** Online, via Qualtrics viewing platform  **Valence:** Pro  **Medium:** Advertisements presented online  **Title:** N/A  **Total duration of presentation:** 310s  **Number of portrayals:** 4  **Duration of portrayals:** 160 s  **Brands presented:** Carling, Jacob’s Creek, WKD, Smirnoff  **Type of alcohol presented:** Lager, wine, alcopop, vodka  **Editing details:** None  **Intervention 2 –**  **Setting:** Online, via Qualtrics viewing platform  **Valence:** Anti  **Medium:** Alcohol warning advertisements presented online  **Title:** N/A  **Total duration of presentation:** 330s  **Number of portrayals:** 4  **Duration of portrayals:** 180 s  **Brands presented:** N/A  **Type of alcohol presented:** N/A  **Editing details:** None  **Control -**  **Setting:** Online, via Qualtrics viewing platform  **Valence:** Anti  **Medium:** Alcohol warning advertisements presented online  **Title:** N/A  **Total duration of presentation:** 310s  **Number of portrayals:** 4  **Duration of portrayals:** 160 s  **Brands presented:** Santander, Barclays, HSBC, Nat West  **Type of alcohol presented:** N/A  **Editing details:** None  **Additional details -**  **Intervention arms:** *Intervention 1* – Alcohol advertising; *Intervention 2* – Alcohol warning advertising*; Control* – Neutral advertising  **Comparisons analysed:** Intervention 1 v Control; Intervention 2 v Control; Intervention 1 v Intervention 2  **Concurrent intervention components:** None |
| **Outcomes** | **Outcomes reported in study:** Explicit alcohol-related cognitions** - Attitudes towards alcohol (**Task:** Questionnaire, **Unit of measurement:** Two items with 7-point response scale);  Implicit alcohol-related cognitions** (**Task:** Implicit Attitudes Test - Alcohol, **Unit of measurement:** Response latency to alcohol+positive compared to alcohol+neutral words);  Alcohol selection without purchasing** (**Task:** Voucher selection – for café or pub, **Unit of measurement:** Dichotomous  **Timing of outcome measurement:** Following experimental manipulation  **Additional outcomes:** None  ** Primary outcome*  *** Secondary Outcome* |
| **Notes** |  |

| **De Graaf (2013)** | |
| --- | --- |
| **Methods** | Randomised, laboratory-based experiment (between-subjects design) |
| **Participants** | **Geographical region:** The Netherlands  **Sampling frame:** High School  **Sampling method:** Not reported  **Number of participants randomised:** 108  **Number (%) of enrolled participants completing the study:** 108 (100%) – Group allocation not reported  **Study completers - Mean age (SD):** 15.34 (0.78)  **Study completers - Sex (Female %):** 45.4  **Ethnicity:** Not reported  **Socio-economic status:** Not reported  **Education:** 60% were in their penultimate years of prevocational education (Level 1), and approximately 40% were in their penultimate year of precollege education (Level 2)  **Baseline alcohol consumption:** N/A  **Executive function:** Not reported  **Inclusion criteria:** Not reported  **Exclusion criteria:** Not reported |
| **Interventions** | **Intervention 1 –**  **Setting:** Not reported  **Valence:** Pro  **Medium:** Television  **Title:** “Jersey Shore”  **Total duration of presentation:** 20 minutes  **Number of portrayals:** Not reported  **Duration of portrayals:** 6 minutes  **Brands presented:** Not reported  **Type of alcohol presented:** Not reported  **Editing details:** Edited to show positive consequences of alcohol consumption  **Intervention 2 –**  **Setting:** Not reported  **Valence:** Anti  **Medium:** Television  **Title:** “Jersey Shore”  **Total duration of presentation:** 20 minutes  **Number of portrayals:** Not reported  **Duration of portrayals:** 6 minutes  **Brands presented:** Not reported  **Type of alcohol presented:** Not reported  **Editing details:** Edited to show negative consequences of alcohol consumption  **Control -**  **Setting:** Not reported  **Valence:** Neutral  **Medium:** Television  **Title:** “Jersey Shore”  **Total duration of presentation:** 20 minutes  **Number of portrayals:** N/A  **Duration of portrayals:** N/A  **Brands presented:** Not reported  **Type of alcohol presented:** Not reported  **Editing details:** Outcome measured prior to presentation of (either) above manipulation  **Additional details -**  **Intervention arms:** *Intervention 1* - Edited film clip showing positive consequences of alcohol consumption only; *Intervention 2* - Edited film clip showing negative consequences of alcohol consumption only; *Control* – Outcome measures prior to presentation of manipulation  **Comparisons analysed:** Intervention 1 v Control; Intervention 2 v Control; Intervention 1 v Intervention 2  **Concurrent intervention components:** None |
| **Outcomes** | **Outcomes reported in study:** Explicit alcohol-related cognitions** - Attitudes/Expectancies towards drinking alcohol (**Task:** Questionnaire; **Unit of measurement:** Five 7-point scales assessing expectancies e.g. *"Drinking alcohol makes you have fun"*; Five 7-point semantic differential scales assessing attitudes e.g. *"I think drinking beer is... Very unpleasant-Very pleasant"*)  **Timing of outcome measurement:** Immediately after the experimental manipulation.  **Additional outcomes:** Engagement with Characters (**Task:** Questionnaire; **Unit of measurement:** Two 7-point scales assessing general engagement e.g. *"During watching, I empathised with the characters of Jersey Shore"*; Two 7-point scales assessing engagement with each individual character e.g. *"I liked Nicole [Snooki]"*)  ** Primary outcome*  *** Secondary Outcome* |
| **Notes** |  |

| **Dunn & Yniguez (1999)** | |
| --- | --- |
| **Methods** | Randomised, school-based experiment (between-subjects design) |
| **Participants** | **Geographical region:** Central Florida county, USA  **Sampling frame:** One of four elementary schools  **Sampling method:** Not reported  **Number of participants randomised:** 551  **Number (%) of enrolled participants completing the study:** 551 (100%) 267 Intervention 1, 284 control  **Study completers - Mean age (SD):** 10.27 (1.04)  **Study completers - Sex (Female %):** 49.18  **Ethnicity:** 67.15 Caucasian; 4.72 African-American; 2.18 Asian-American; 20.87 Hispanic; 4.72 other  **Socio-economic status:** Not reported  **Education:** Fourth-Fifth grade children  **Baseline alcohol consumption:** N/A  **Executive function:** Not reported  **Inclusion criteria:** Not reported  **Exclusion criteria:** Not reported |
| **Interventions** | **Intervention 1 –**  **Setting:** Classroom  **Valence:** Pro  **Medium:** Television (Advertisements)  **Title:** Not reported  **Total duration of presentation:** Not reported  **Number of portrayals:** 5  **Duration of portrayals:** Not reported  **Brands presented:** Not reported  **Type of alcohol presented:** Beer  **Editing details:** None  **Control -**  **Setting:** Classroom  **Valence:** Neutral  **Medium:** Television (Advertisements)  **Title:** Not reported  **Total duration of presentation:** Not reported  **Number of portrayals:** 5  **Duration of portrayals:** Not reported  **Brands presented:** Not reported  **Type of alcohol presented:** Soft drink  **Editing details:** None  **Additional details -**  **Intervention arms:** *Intervention 1* – Alcohol advertisements; *Control* – Soft drink advertisements  **Comparisons analysed:** Intervention 1 v Control  **Concurrent intervention components:** None |
| **Outcomes** | **Outcomes reported in study:** Explicit alcohol-related cognitions** - Expectancies (**Task:** Questionnaire - Adjectives rated for how often they are experienced during alcohol consumption [Dunn & Goldman, 1996]; **Unit of Measurement:** 4-point scale, Number of items not reported)  **Timing of outcome measurement:** Immediately following the experimental manipulation  **Additional outcomes:** Self reported **a**lcoholic beverage consumption (**Task:** Children's drinking habits questionnaire [Christiansen & Goldman, 1983]; **Unit of Measurement:** Percentage of sample engaging in drinking behaviour **[**Quantity/frequency measure consisting of yes/no responses])  ** Primary outcome*  *** Secondary Outcome* |
| **Notes** |  |

| **Engels, Hermans, van Baaren, Hollenstein, & Bot (2009)** | |
| --- | --- |
| **Methods** | Randomised, laboratory-based experiment (between-subjects design) |
| **Participants** | **Sampling frame:** Radboud University Nijmegen  **Geographical region:** Netherlands  **Sampling method:** Opportunity (Flyers & course credit)  **Number of participants randomised:** 80  **Number (%) of enrolled participants completing the study:** 80 (100%) 40 Intervention 1, 40 control  **Study completers - Mean age (SD):** 21.45 (2.19)  **Study completers - Sex (Female %):** 0  **Ethnicity:** Not reported  **Socio-economic status context:** Not reported  **Education:** A-Level (Level 3) - Inferred from undergraduate student status  **Baseline alcohol consumption:** Average weekly consumption 21.05 (15.12) glasses.  **Executive function:** Not reported  **Inclusion criteria:** Males  **Exclusion criteria:** Not reported |
| **Interventions** | **Intervention 1 –**  **Setting:** Bar laboratory  **Valence:** Pro  **Medium:** Film  **Title:** “American Pie 2”  **Total duration of presentation:** 60 minutes  **Number of portrayals:** 41  **Duration of portrayals:** Not reported  **Brands presented:** Not reported  **Type of alcohol presented:** Not reported  **Editing details:** Not reported  **Intervention 2 –**  **Setting:** Bar laboratory  **Valence:** Pro  **Medium:** Television (Advertisements)  **Title:** Not reported  **Total duration of presentation:** 3.5 minutes  **Number of portrayals:** 2  **Duration of portrayals:** Not reported  **Brands presented:** Not reported  **Type of alcohol presented:** Not reported  **Editing details:** Not reported  **Control 1 -**  **Setting:** Bar laboratory  **Valence:** Neutral  **Medium:** Film  **Title:** “40 days and 40 nights”  **Total duration of presentation:** 60 minutes  **Number of portrayals:** 18 (alcohol)  **Duration of portrayals:** Not reported  **Brands presented:** Not reported  **Type of alcohol presented:** Not reported  **Editing details:** None  **Control 2 -**  **Setting:** Bar laboratory  **Valence:** Neutral  **Medium:** Television (Advertisements)  **Title:** Not reported  **Total duration of presentation:** 7 minutes  **Number of portrayals:** Not reported  **Duration of portrayals:** Not reported  **Brands presented:** Not reported  **Type of alcohol presented:** Not reported  **Editing details:** None  **Additional details -**  **Intervention arms:** *Intervention 1* – Film with numerous alcohol portrayals; *Intervention 2* – Alcohol advertisements; *Control* 1- Film with less alcohol portrayals ; Control 2 – Non-alcohol advertisements  **Comparisons analysed:** Intervention 1 v Control 1; Intervention 2 v Control 2; Intervention 1 v Intervention 2  **Concurrent intervention components:** None |
| **Outcomes** | **Outcomes reported in study:** Alcoholic beverage consumption* (**Task:** Alcohol consumption over the course of a film; **Unit of Measurement:** Number of [beer] bottles consumed)  **Timing of outcome measurement:** During the experimental manipulation  **Additional outcomes:** Appreciation of the film (**Task:** Questionnaire; **Unit of measurement:** Nine 5-point scale items e.g. *"I liked the movie"*, *" I appreciated the theme of the movie"*); Familiarity with the film (**Task:** Single item question; **Unit of measurement:** Dichotomous answer of whether they has seen the film before or not); Self reported **a**lcoholic beverage consumption (**Task:** Questionnaire; **Unit of measurement:** Quantity/frequency measures including how many drinks are commonly consumed when watching an hour of TV, frequency of drinking over the past 12 months; total consumption over the past 7 days)  ** Primary outcome*  *** Secondary Outcome* |
| **Notes** |  |

| **Goodall & Slater (2010)** | |
| --- | --- |
| **Methods** | Randomised, laboratory-based experiment (between-subjects design) |
| **Participants** | **Geographical region:** USA  **Sampling frame:** University  **Sampling method:** Opportunity sampling (course credit)  **Number of participants randomised:** 145  **Number (%) of enrolled participants completing the study:** 145 (100%) 48 Intervention 1, 47 intervention 2, 47 control  **Study completers - Mean age (SD):** Not reported - College students  **Study completers - Sex (Female %):** Not reported  **Ethnicity:** Not reported  **Socio-economic status:** Not reported  **Education:** A-Level (Level 3) - Inferred from undergraduate student status  **Baseline alcohol consumption:** Not reported  **Executive function:** Not reported  **Inclusion criteria:** Not reported  **Exclusion criteria:** Individuals who speak Chinese or Japanese |
| **Interventions** | **Intervention 1 –**  **Setting:** Computer laboratory  **Valence:** Pro  **Medium:** Television (Advertisements)  **Title:** Not reported  **Total duration of presentation:** 2 minutes  **Number of portrayals:** 4  **Duration of portrayals:** Not reported  **Brands presented:** Bacardi; Budweiser; Heineken; Miller  **Type of alcohol presented:** Beer, Vodka  **Editing details:** None  **Intervention 2 –**  **Setting:** Computer laboratory  **Valence:** Anti  **Medium:** Television (Advertisements)  **Title:** Not reported  **Total duration of presentation:** 2 minutes  **Number of portrayals:** 4  **Duration of portrayals:** Not reported  **Brands presented:** Government PSAs: Mothers against drunk driving; Ad council  **Type of alcohol presented:** Not reported  **Editing details:** None  **Control -**  **Setting:** Computer laboratory  **Valence:** Neutral  **Medium:** Television (Advertisements)  **Title:** Not reported  **Total duration of presentation:** 2 minutes  **Number of portrayals:** 4  **Duration of portrayals:** Not reported  **Brands presented:** Apple computers; AT&T; Nike; Southwest Airlines  **Type of alcohol presented:** N/A  **Editing details:** None  **Additional details -**  **Intervention arms:** *Intervention 1* – Alcohol advertisements; *Intervention 2* – Anti-alcohol advertisements; *Control* – Neutral advertisements  **Comparisons analysed:** Intervention 1 v Control; Intervention 2 v Control; Intervention 1 v Intervention 2  **Concurrent intervention components:** None |
| **Outcomes** | **Outcomes reported in study:** Implicit alcohol-related cognitions** - Attitudes (**Task:** Affective misattribution procedure**; Unit of measurement:** Proportion of pleasant/favourable responses);  Explicit alcohol-related cognitions** - Attitudes (**Task:** Questionnaire**, Unit of measurement:** Average response from 10-point Semantic differential assessing attitudes towards beer, liqour/mixed drinks and wine)  **Timing of outcome measurement:** Immediately after the experimental manipulation  **Additional outcomes:** Self reported alcoholic beverage consumption (**Task:** Questionnaire**; Unit of measurement:** Frequency of drinking [5- point scale] and quantity on those occasions averaged into a single item index); Behavioural willingness (**Task:** Questionnaire [Gibbons et al. 2003]**; Unit of measurement:** Single item index - derived from five 10-point semantic differential responses to two vignettes. Items were factor analysed and reduced to form the single index); Message evaluation (**Task:** Questionnaire**; Unit of measurement:** Three 10-point semantic differentials indicating how enjoyable, likable and appealing the advert manipulations were)  ** Primary outcome*  *** Secondary Outcome* |
| **Notes** | Numbers of participants incorrectly reported in article. |

| **Kohn & Smart (1984)** | |
| --- | --- |
| **Methods** | Randomised, laboratory-based experiment (between-subjects design) |
| **Participants** | **Geographical region:** Toronto, Canada  **Sampling frame:** York University  **Sampling method:** Opportunity sample (Introductory psychology students)  **Number of participants randomised:** 125  **Number (%) of enrolled participants completing the study:** 125 (100%) 38 Intervention 1, 39 intervention 2, 48 control  **Study completers - Mean age (SD):** Not reported  **Study completers - Sex (Female %):** 0  **Ethnicity:** Not reported  **Socio-economic status:** Not reported  **Education:** A-Level (Level 3) - Inferred from undergraduate student status  **Baseline alcohol consumption:** Not reported  **Executive function:** Not reported  **Inclusion criteria:** Male  **Exclusion criteria:** Not reported |
| **Interventions** | **Intervention 1 –**  **Setting:** Enclosed simulated bar environment, with comfortable overstuffed chairs and subdued lighting  **Valence:** Pro  **Medium:** Television (Advertisements)  **Title:** Not reported  **Total duration of presentation:** 4.5 minutes  **Number of portrayals:** 9  **Duration of portrayals:** Not reported  **Brands presented:** Not reported  **Type of alcohol presented:** Not reported  **Editing details:** Not reported  **Intervention 2 –**  **Setting:** Enclosed simulated bar environment, with comfortable overstuffed chairs and subdued lighting  **Valence:** Pro  **Medium:** Television (Advertisements)  **Title:** N/A  **Total duration of presentation:** 1.5 minutes  **Number of portrayals:** 4  **Duration of portrayals:** Not reported  **Brands presented:** Not reported  **Type of alcohol presented:** Not reported  **Editing details:** Not reported  **Control -**  **Setting:** Enclosed simulated bar environment, with comfortable overstuffed chairs and subdued lighting  **Valence:** Neutral  **Medium:** Television (Advertisements)  **Title:** Not reported  **Total duration of presentation:** 4.5 minutes  **Number of portrayals:** 9  **Duration of portrayals:** Not reported  **Brands presented:** Not reported  **Type of alcohol presented:** None  **Editing details:** Not reported  **Additional details -**  **Intervention arms:** *Intervention 1* – Alcohol adverts (9); *Intervention 2* – Alcohol adverts (4); *Control* – Neutral adverts  **Comparisons analysed:** Intervention 1 v Control; Intervention 2 v Control; Intervention 1 v Intervention 2  **Concurrent intervention components:** None |
| **Outcomes** | **Outcomes reported in study:** Alcoholic beverage consumption* (**Task:** Number of beverage consumed during TV sports game**; Unit of measurement:** Number of beers consumed)  **Timing of outcome measurement:** During the experimental manipulation/(30 minute) Delay, according to experimental condition  **Additional outcomes:** Not reported  ** Primary outcome*  *** Secondary Outcome* |
| **Notes** |  |

| **Kohn & Smart (1987)** | |
| --- | --- |
| **Methods** | Randomised, laboratory-based experiment (between-subjects design) |
| **Participants** | **Geographical region:** Toronto, Canada  **Sampling frame:** York University campus  **Sampling method:** Opportunity sample (Introductory psychology students)  **Number of participants randomised:** 66  **Number (%) of enrolled participants completing the study:** 66 (100%) 23 Intervention 1, 22 intervention 2, 21 control  **Study completers - Mean age (SD):** Not reported  **Study completers - Sex (Female %):** 100  **Ethnicity:** Not reported  **Socio-economic status:** Not reported  **Education:** A-Level (Level 3) - Inferred from undergraduate student status  **Baseline alcohol consumption:** Not reported  **Executive function:** Not reported  **Inclusion criteria:** Female  **Exclusion criteria:** Not reported |
| **Interventions** | **Intervention 1 –**  **Setting:** Enclosed simulated bar environment, with comfortable overstuffed chairs and subdued lighting  **Valence:** Pro  **Medium:** Television (Advertisements)  **Title:** Not reported  **Total duration of presentation:** 4.5 minutes  **Number of portrayals:** 9  **Duration of portrayals:** Not reported  **Brands presented:** Not reported  **Type of alcohol presented:** Wine  **Editing details:** Not reported  **Intervention 2 –**  **Setting:** Enclosed simulated bar environment, with comfortable overstuffed chairs and subdued lighting  **Valence:** Pro  **Medium:** Television (Advertisements)  **Title:** N/A  **Total duration of presentation:** 1.5 minutes  **Number of portrayals:** 4  **Duration of portrayals:** Not reported  **Brands presented:** Not reported  **Type of alcohol presented:** Not reported  **Editing details:** Not reported  **Control -**  **Setting:** Enclosed simulated bar environment, with comfortable overstuffed chairs and subdued lighting  **Valence:** Neutral  **Medium:** Television (Advertisements)  **Title:** Not reported  **Total duration of presentation:** 4.5 minutes  **Number of portrayals:** 9  **Duration of portrayals:** Not reported  **Brands presented:** Not reported  **Type of alcohol presented:** None  **Editing details:** Not reported  **Additional details -**  **Intervention arms:** *Intervention 1* – Alcohol adverts (9); *Intervention 2* – Alcohol adverts (4); *Control* – Neutral adverts  **Comparisons analysed:** Intervention 1 v Control; Intervention 2 v Control; Intervention 1 v Intervention 2  **Concurrent intervention components:** None |
| **Outcomes** | **Outcomes reported in study:** Alcoholic beverage consumption* (**Task:** Number of beverages consumed during TV programming**; Unit of measurement:** Number of glasses of wine consumed [square root transformed])  **Timing of outcome measurement:** During the experimental manipulation  **Additional outcomes:** Awareness questionnaire [Kohn & Barnes, 1977] (**Task:** Questionnaire - 3 items, coded for overall suspiciousness of study aims **Unit of measurement:** Open ended responses)  ** Primary outcome*  *** Secondary Outcome* |
| **Notes** |  |

| **Koordeman, Anschutz, & Engels (2011)** | |
| --- | --- |
| **Methods** | Randomised, laboratory-based experiment (between-subjects design) |
| **Participants** | **Geographical region:** The Netherlands  **Sampling frame:** Radboud university campus  **Sampling method:** Opportunity sample (Posters & Flyers)  **Number of participants randomised:** 184  **Number (%) of enrolled participants completing the study:** 184 (100%) - Group allocation not reported  **Study completers - Mean age (SD):** 22 (3.3)  **Study completers - Sex (Female %):** 50  **Ethnicity:** Not reported  **Socio-economic status:** Not reported  **Education:** 80% A-Level (Level 3) - Inferred from undergraduate student status  **Baseline alcohol consumption:** Not reported  **Executive function:** Not reported  **Inclusion criteria:** Aged > 16  **Exclusion criteria:** Not reported |
| **Interventions** | **Intervention 1 –**  **Setting:** “Serving cinema” at Radboud University Campus  **Valence:** Pro  **Medium:** Television (Adverts)  **Title:** “Barry Harry” (Grolsch); “Release the beast/Dungeon” (Jagermeister); “Land of the Wolves” (Bacardi Eristoff); “Muddle” (Bacardi Mojito)  **Total duration of presentation:** 5 minutes, 20 seconds  **Number of portrayals:** 4  **Duration of portrayals:** Not reported  **Brands presented:** Grolsch; Jagermeister; Bacardi  **Type of alcohol presented:** Beer, Spirits  **Editing details:** Adverts shown during the film “Watchmen”  **Control –**  **Setting:** “Serving cinema” at Radboud University Campus  **Valence:** Neutral  **Medium:** Television (Adverts)  **Title:** “Houseparty” (Adidas); Tourism Australia/Malaysia airlines; “Axe Primate” (Axe); Jean Mineur Mediavision generique debut; NVB credit note; “KillZone” (Sony Playstation)  **Total duration of presentation:** 3 minutes, 20 seconds  **Number of portrayals:** Not reported  **Duration of portrayals:** Not reported  **Brands presented:** Adidas; Tourism Australia/Malaysia airlines; Axe; Jean Mineur Mediavision generique debut; NVB; Sony Playstation  **Type of alcohol presented:** None  **Editing details:** Adverts shown during the film “Watchmen”  **Additional details -**  **Intervention arms:** *Intervention 1 -* Alcohol advertisements; *Control* – Neutral Adverts  **Comparisons analysed:** Intervention 1 v Control  **Concurrent intervention components:** None |
| **Outcomes** | **Outcomes reported in study:** Alcoholic beverage consumption* (**Task:** Alcohol consumption during a film**; Unit of measurement:** Number of bottles & cl consumed)  **Timing of outcome measurement:** During the experimental manipulation  **Additional outcomes:** Self reported alcoholic beverage consumption (**Task:** Questionnaire; **Unit of measurement:** Single item Quantity/frequency measure - consumption over the previous 7 days and how much was consumed; Two-item measure of problem alcohol use: *"In the past 12 months have you tried to stop drinking without suceeding?" "In the past 12 months have you consumed alcohol to forget your problems?"*); Attitude towards the commercials (**Task:** Questionnaire **Unit of measurement:** Nine 5-point scales e.g. *"I liked the kind of ads I've seen"*, *"I felt better after seeing the ads"* ); Attitude towards the film (**Task:** Questionnaire, **Unit of measurement:** Six 5-point scales e.g. *"I found it a boring movie"*, *"I liked the movie"*)  ** Primary outcome*  *** Secondary Outcome* |
| **Notes** |  |

| **Koordeman, Anschutz, van Baaren, & Engels (2011)** | |
| --- | --- |
| **Methods** | Randomised, laboratory-based experiment (between-subjects design) |
| **Participants** | **Geographical region:** The Netherlands  **Sampling frame:** Radboud University Nijmegen  **Sampling method:** Opportunity sample (Flyers and course credit)  **Number of participants randomised:** 244  **Number (%) of enrolled participants completing the study:** 244 (100%) – Group allocation not reported  **Study completers - Mean age (SD):** 21 (2.54)  **Study completers - Sex (Female %):** 54  **Ethnicity:** Not reported  **Socio-economic status:** Not reported  **Education:** A-Level (Level 3) - Inferred from undergraduate student status  **Baseline alcohol consumption:** Not reported  **Executive function:** Not reported  **Inclusion criteria:** Not reported  **Exclusion criteria:** Not reported |
| **Interventions** | **Intervention 1 –**  **Setting:** Semi-naturalistic setting in a laboratory room with relaxing couches and decorations  **Valence:** Pro  **Medium:** Film  **Title:** “What Happens In Vegas”  **Total duration of presentation:** 60 minutes  **Number of portrayals:** 18  **Duration of portrayals:** 9 minutes, 25 seconds  **Brands presented:** Not reported  **Type of alcohol presented:** Not reported  **Editing details:** Edited to contain alcohol portrayals – “Alcohol portrayal was defined as real or implied used of alcohol, including occasions where an alcoholic beverage was clearly in the possession of a character or mentioned verbally. Alcoholic beverages that were displayed, but not implied as being consumed by a character were not coded as an alcohol portrayal.”  **Control -**  **Setting:** Semi-naturalistic setting in a laboratory room with relaxing couches and decorations  **Valence:** Neutral  **Medium:** Film  **Title:** “What happens in vegas”  **Total duration of presentation:** 60 minutes  **Number of portrayals:** Not reported  **Duration of portrayals:** Not reported  **Brands presented:** Not reported  **Type of alcohol presented:** Not reported  **Editing details:** Edited to show exclusively non-alcoholic scenes  **Additional details -**  **Intervention arms:** *Intervention 1* – Film edited to contain alcohol portrayals; *Control* – Film edited to show exclusively non-alcoholic scenes  **Comparisons analysed:** Intervention 1 v Control  **Concurrent intervention components:** None |
| **Outcomes** | **Outcomes reported in study:** Alcoholic beverage consumption* (**Task:** Alcohol consumption over the course of a film, **Unit of measurement:** Number of bottles & cl consumed)  **Timing of outcome measurement:** During the experimental manipulation  **Additional outcomes:** Self reported alcoholic beverage consumption (**Task:** Questionnaire; **Unit of measurement:** Single item Quantity/frequency measure - consumption over the previous 7 days and how much was consumed; Two-item measure of problem alcohol use: *"In the past 12 months have you tried to stop drinking without suceeding?" "In the past 12 months have you consumed alcohol to forget your problems?"*); Attitude towards the film (**Task:** Questionnaire, **Unit of measurement:** Six 5-point scales e.g. *"I found it a boring movie"*, *"I liked the movie"*)  ** Primary outcome*  *** Secondary Outcome* |
| **Notes** |  |

| **Koordeman, Anschutz, & Engels (2012)** | |
| --- | --- |
| **Methods** | Randomised, laboratory-based experiment (between-subjects design) |
| **Participants** | **Geographical region:** The Netherlands  **Sampling frame:** Radboud University  **Sampling method:** Opportunity (Flyers and course credit)  **Number of participants randomised:** 160  **Number (%) of enrolled participants completing the study:** 159 (99%) 80 Intervention, 79 control  **Study completers - Mean age (SD):** 21.08 (2.7)  **Study completers - Sex (Female %):** 0  **Ethnicity:** Not reported  **Socio-economic status:** Not reported  **Education:** 50% A-Level (Level 3) At least 50% university students - 50% of the sample were recruited from university and asked to bring a friend.  **Baseline alcohol consumption:** 15.90 (15 cl) glasses per week  **Executive function:** Not reported  **Inclusion criteria:** Not reported  **Exclusion criteria:** Not reported |
| **Interventions** | **Intervention 1 –**  **Setting:** University ("semi-naturalistic") laboratory setting  **Valence:** Pro  **Medium:** Television (Advertisements)  **Title:** Not reported  **Total duration of presentation:** 60 minutes  **Number of portrayals:** 6  **Duration of portrayals:** 2.5 minutes  **Brands presented:** Amstel beer, Bacardi mojito, Brand beer, Jillz sparkling cider, Martini ice tea, Warsteiner beer  **Type of alcohol presented:** Beer, Cider, Cocktail, Spirits  **Editing details:** Interspersed at 11, 28 & 43 minutes of a 60 minute film clip of “Planet Earth”  **Control -**  **Setting:** University ("semi-naturalistic") laboratory setting  **Valence:** Neutral  **Medium:** Television (Advertisements)  **Title:** Not reported  **Total duration of presentation:** 60 minutes  **Number of portrayals:** 5  **Duration of portrayals:** 2.5 minutes  **Brands presented:** Not reported  **Type of alcohol presented:** None  **Editing details:** Interspersed at 11, 28 & 43 minutes of a 60 minute film clip of “Planet Earth”  **Additional details -**  **Intervention arms:** *Intervention 1* – Alcohol advertisements; *Control* – Neutral advertisements  **Comparisons analysed:** Intervention 1 v Control  **Concurrent intervention components:** None |
| **Outcomes** | **Outcomes reported in study:** Alcoholic Beverage Consumption* (**Task:** Alcohol consumption whilst watching a film; **Unit of measurement**: Amount of bottles & Cl consumed); Alcohol expectancies** - Positive and arousal based (**Task:** Questionnaire; **Unit of measurement:** Two 6-item semantic differential scales - "Drinking makes me"... *fun, happy, impulsive, energetic*.)  **Timing of outcome measurement:** Consumption - During manipulation; Expectancies - Immediately after manipulation.  **Additional outcomes:** Recall and attitudes towards the adverts (**Task:** Questionnaire; **Unit of measurement:** Nine 5-point scales - e.g. "*I found the ads interesting*", "*I was aware of the presented products in the ads*"); Scepticism (**Task:** Questionnaire; **Unit of measurement:** 4 point scales - e.g. *"How often do you think an advertisement is honest?"*, *"How often do you believe in advertisement message?"*)  ** Primary outcome*  *** Secondary Outcome* |
| **Notes** |  |

| **Koordeman, Anschutz, & Engels (2015)** | |
| --- | --- |
| **Methods** | Randomised, laboratory-based experiment (between-subjects design) |
| **Participants** | **Geographical region:** The Netherlands  **Sampling frame:** Radboud University  **Sampling method:** Opportunity (Flyers, internet advertising, and course credit)  **Number of participants randomised:** 154  **Number (%) of enrolled participants completing the study:** 154 (100%)  **Study completers - Mean age (SD):** 21.4 (2.57)  **Study completers - Sex (Female %):** 0  **Ethnicity:** Not reported  **Socio-economic status:** Not reported  **Education:** A-Level (Level 3) - Inferred from undergraduate student status  **Baseline alcohol consumption:** Mean of 17.36 (SD=14.26) alcoholic beverages consumed in past week  **Executive function:** Assessed self control with a questionnaire – mean not reported  **Inclusion criteria:** Not reported  **Exclusion criteria:** Not reported |
| **Interventions** | **Intervention 1 –**  **Setting:** University laboratory setting – “designed to resemble a living room”  **Valence:** Pro  **Medium:** Film  **Title:** “Get Him To The Greek”  **Total duration of presentation:** 60 minutes  **Number of portrayals:** 17  **Duration of portrayals:** 490 s  **Brands presented:** Not reported  **Type of alcohol presented:** Not reported  **Editing details:** “Two versions of the movie were created; one showing scenes containing alcohol portrayal in addition to non-alcoholic scenes and the other showing exclusively non-alcoholic scenes”  **Control -**  **Setting:** University laboratory setting – “designed to resemble a living room”  **Valence:** Neutral  **Medium:** Film  **Title:** “Get Him To The Greek”  **Total duration of presentation:** 60 minutes  **Number of portrayals:** 0  **Duration of portrayals:** N/A  **Brands presented:** N/A  **Type of alcohol presented:** N/A  **Editing details:** “Two versions of the movie were created; one showing scenes containing alcohol portrayal in addition to non-alcoholic scenes and the other showing exclusively non-alcoholic scenes”  **Additional details -**  **Intervention arms:** *Intervention 1* – Alcohol portrayals; *Control* – No alcohol portrayals  **Comparisons analysed:** Intervention 1 v Control  **Concurrent intervention components:** None |
| **Outcomes** | **Outcomes reported in study:** Alcoholic Beverage Consumption* (**Task:** Alcohol consumption whilst watching a film; **Unit of measurement**: Amount of bottles & Cl consumed);  **Timing of outcome measurement:** During manipulation  **Additional outcomes:** Evaluation of the movie.  ** Primary outcome*  *** Secondary Outcome* |
| **Notes** |  |

| **Kotch, Coulter, & Lipsitz (1986)** | |
| --- | --- |
| **Methods** | Randomised, school-based experiment (between-subjects design) |
| **Participants** | **Geographical region:** North Carolina, USA  **Sampling frame:** Public elementary school  **Sampling method:** Opportunity sample - Letters sent to parents  **Number of participants randomised:** 61  **Number (%) of enrolled participants completing the study:** 43 (70%) - Group allocation not reported  **Study completers - Mean age (SD):** Not reported - 19 fifth graders, 24 sixth graders  **Study completers - Sex (Female %):** 56  **Ethnicity:** Not reported  **Socio-economic status:** Not reported  **Education:** 5th-6th grade  **Baseline alcohol consumption:** N/A  **Executive function:** Not reported  **Inclusion criteria:** Not reported  **Exclusion criteria:** Not reported |
| **Interventions** | **Intervention 1 –**  **Setting:** Not reported  **Valence:** Pro  **Medium:** Television  **Title:** Not reported  **Total duration of presentation:** 35 minutes  **Number of portrayals:** 35  **Duration of portrayals:** Not reported  **Brands presented:** Not reported  **Type of alcohol presented:** Not reported  **Editing details:** Edited to contain scenes in which characters drank an alcoholic beverage without negative consequences  **Control -**  **Setting:** Not reported  **Valence:** Neutral  **Medium:** Television  **Title:** Not reported  **Total duration of presentation:** 35 minutes  **Number of portrayals:** Not reported  **Duration of portrayals:** Not reported  **Brands presented:** Not reported  **Type of alcohol presented:** N/A  **Editing details:** Edited to contain scenes in which no drinking occurred.  **Additional details -**  **Intervention arms:** *Intervention 1* – TV programme edited to contain alcohol consumption scenes; *Control* - TV programme edited to contain scenes with no alcohol consumption  **Comparisons analysed:** Intervention 1 v Control  **Concurrent intervention components:** None |
| **Outcomes** | **Outcomes reported in study:** Explicit alcohol-related cognitions** (**Task:** Expectancy questionnaire - Subjective utility scale [Bauman & Bryan, 1980]; **Unit of measurement:** 45 questions about the salience and probability of alcohol related outcomes. Response format not reported.); Explicit alcohol-related cognitions** (**Task:** Attitude questionnaire - "How wrong is it" [Gorsuch & Arno, 1979]; **Unit of measurement:** Twenty four 5-point questions about approval/disapproval of certain alcohol consumption situations)  **Timing of outcome measurement:** Immediately following the experimental manipulation  **Additional outcomes:** None reported  ** Primary outcome*  *** Secondary Outcome* |
| **Notes** | Indirect measure of consumption |

| **Kulick & Rosenberg (2006)** | |
| --- | --- |
| **Methods** | Randomised, laboratory-based experiment (between-subjects design) |
| **Participants** | **Geographical region:** USA  **Sampling frame:** University  **Sampling method:** Opportunity (course credit)  **Number of participants randomised:** 116  **Number (%) of enrolled participants completing the study:** 108 (93%). 37 intervention 1; 37 intervention 2; 34 control.  **Study completers - Mean age (SD):** 18.42  **Study completers - Sex (Female %):** 70.37  **Ethnicity:** 94 Caucasian, 5 African American, 4 Hispanic, 5  did not indicate their ethnicity  **Socio-economic status:** Not reported  **Education:** All but 2 were in first year of college  **Baseline alcohol consumption:** 9 abstinent, 92 occasional or moderate social drinkers, 7 frequent heavy social drinkers  **Executive function:**  **Inclusion criteria:**  **Exclusion criteria:** Participants aged over 19 excluded |
| **Interventions** | **Intervention 1 –**  **Setting:** University  **Valence:** Pro  **Medium:** Film  **Title:** Not reported  **Total duration of presentation:** 51 minutes, 38 seconds  **Number of portrayals:** 6 (plus 2 non-alcohol portrayals) viewed twice  **Duration of portrayals:** 41 minutes, 22 seconds  **Brands presented:** Not reported  **Type of alcohol presented:** Not reported  **Editing details:** Film clips depicted “desirable outcomes such as laughing, singing, dancing, and companionship”  **Intervention 2 –**  **Setting:** University  **Valence:** Anti  **Medium:** Film  **Title:** Not reported  **Total duration of presentation:** 53 minutes, 24 seconds  **Number of portrayals:** 5 (plus 3 non-alcohol portrayals) viewed twice  **Duration of portrayals:** 43 minutes, 24 seconds  **Brands presented:** Not reported  **Type of alcohol presented:** Not reported  **Editing details:** Film clips depicted “undesirable outcomes such as aggressive and violent behaviour, vomiting, and depressed mood”  **Control -**  **Setting:** University  **Valence:** Neutral  **Medium:** Film  **Title:** Not reported  **Total duration of presentation:** 49 minutes, 36 seconds  **Number of portrayals:** 8, viewed twice  **Duration of portrayals:** 49 minutes, 36 seconds  **Brands presented:** Not reported  **Type of alcohol presented:** N/A  **Editing details:** Film clips containing scenes in which individuals did not consume alcohol  **Additional details -**  **Intervention arms:** *Intervention 1* –Film clips with alcohol consumption and positive consequences; *Control* – Film clips with no alcohol consumption  **Comparisons analysed:** Intervention 1 v Control  **Concurrent intervention components:** None |
| **Outcomes** | **Outcomes reported in study:** Outcome expectancies** (**Task:** Comprehensive Effects of Alcohol scale (Fromme, Stroot, & Kaplan, 1993);**Unit of measurement:** 38 items – summary scores generated for positive and negative expectancies)  **Timing of outcome measurement:** Immediately following the experimental manipulation.  **Additional outcomes:** Intentions to drink alcohol in the next week. (**Task**: three self-report items asking participants to report how likely they were to drink beer, wine, and distilled spirits in the next week if drinking age restrictions did not exist; **Unit of measurement**: Mean score between 1 and 4).  ** Primary outcome*  *** Secondary Outcome* |
| **Notes** |  |

| **McCarty & Ewing (1983)** | |
| --- | --- |
| **Methods** | Randomised, laboratory-based experiment (between-subjects design) |
| **Participants** | **Geographical region:** USA  **Sampling frame:** Not reported  **Sampling method:** Opportunity sample (Newspapers & Posters)  **Number of participants randomised:** 112  **Number (%) of enrolled participants completing the study:** 112 (100%) 56 Intervention 1, 56 control  **Study completers - Mean age (SD):** Not reported  **Study completers - Sex (Female %):** 57  **Ethnicity:** Not reported  **Socio-economic status:** Not reported  **Education:** Not reported  **Baseline alcohol consumption:** 40% heavy drinkers, 36% moderate drinkers, 24 light drinkers (Quantity frequency classification: Cahalan, Cisin & Crossley, 1996)  **Executive function:** Not reported  **Inclusion criteria:** Not reported  **Exclusion criteria:** Not reported |
| **Interventions** | **Intervention 1 –**  **Setting:** Not reported  **Valence:** Pro  **Medium:** Photographic slides  **Title:** Not reported  **Total duration of presentation:** 30 minutes  **Number of portrayals:** 8  **Duration of portrayals:** 24 minutes  **Brands presented:** Not reported  **Type of alcohol presented:** Bourbon, Brady, Scotch, Whisky, Gin, Liquor, Vodka  **Editing details:** Not reported  **Control -**  **Setting:** Not reported  **Valence:** Neutral  **Medium:** Photographic slides  **Title:** Not reported  **Total duration of presentation:** 30 minutes  **Number of portrayals:** 10  **Duration of portrayals:** 30 minutes  **Brands presented:** Not reported  **Type of alcohol presented:** Overcoats, sour cream, cologne, perfume, furniture, car, hair dye, lipstick  **Editing details:** Not reported  **Additional details -**  **Intervention arms:** *Intervention 1* – Alcohol-related slides; *Intervention 2* - ; *Control* – Neutral slides  **Comparisons analysed:** Intervention 1 v Control  **Concurrent intervention components:** None |
| **Outcomes** | **Outcomes reported in study:** Alcoholic beverage consumption* (**Task:** Alcohol consumption whilst watching advertisements; **Unit of measurement:** ml left after consumption)  **Timing of outcome measurement:** During experimental manipulation  **Additional outcomes:** Breath alcohol concentration (**Task:** Breathalyser; **Unit of measurement:** mg %)  ** Primary outcome*  *** Secondary Outcome* |
| **Notes** |  |

| **Roehrich & Goldman (1995)** | |
| --- | --- |
| **Methods** | Randomised, laboratory-based experiment (between-subjects design) |
| **Participants** | **Sampling frame:** University of South Florida  **Geographical region:** Florida, USA  **Sampling method:** Opportunity (Introductory class for course credit)  **Number of participants randomised:** 94  **Number (%) of enrolled participants completing the study:** 80 (85%) 40 Intervention 1, 40 control  **Study completers - Mean age (SD):** 25.25 (Not reported)  **Study completers - Sex (Female %):** 100  **Ethnicity:** Not reported  **Socio-economic status:** Not reported  **Education:** A-Level (Level 3) - Inferred from undergraduate student status  **Baseline alcohol consumption:** 5.58 drinking episodes per month; 1-2 drinks per occasion  (Quantity frequency classification: Cahalan, Cisin & Crossley, 1996)  **Executive function:** Not reported  **Inclusion criteria:** Female  **Exclusion criteria:** Not reported |
| **Interventions** | **Intervention 1 –**  **Setting:** “Psychology laboratory that contained several desks and chairs, a video recorder, a television monitor and a slide projector”  **Valence:** Pro  **Medium:** Television  **Title:** “Cheers”  **Total duration of presentation:** 3.5 minutes  **Number of portrayals:** Not reported  **Duration of portrayals:** Not reported  **Brands presented:** Not reported  **Type of alcohol presented:** Not reported  **Editing details:** Not reported  **Control -**  **Setting:** “Psychology laboratory that contained several desks and chairs, a video recorder, a television monitor and a slide projector”  **Valence:** Neutral  **Medium:** Television  **Title:** “Newhart”  **Total duration of presentation:** 3.5 minutes  **Number of portrayals:** Not reported  **Duration of portrayals:** Not reported  **Brands presented:** Not reported  **Type of alcohol presented:** Not reported  **Editing details:** Not reported  **Additional details -**  **Intervention arms:** *Intervention 1* – Alcohol related TV programme; *Control* - Neutral TV programme  **Comparisons analysed:** Intervention 1 v Control  **Concurrent intervention components:** Priming (Alcohol expectancy, Neutral) |
| **Outcomes** | **Outcomes reported in study:** Alcoholic beverage consumption* (**Task:** Beer "Taste test" [Marlatt et al. 1973]; **Unit of measurement:** ml)  **Timing of outcome measurement:** Immediately following experimental manipulation  **Additional outcomes:** Self reported alcoholic beverage consumption (**Task:** Quantity, Frequency and Variability index [Cahalan, Cisley & Crossley, 1969]; **Unit of measurement:** Quantity of alcohol consumed in each episode, Frequency of consumption episodes); Word recognition test (**Task:** Test for memory of word presented during Stroop priming manipulation; **Unit of measurement:** Number of words remembered)  ** Primary outcome*  *** Secondary Outcome* |
| **Notes** |  |

| **Rychtarik, Fairbank, Allen, Foy, & Drabman (1983)** | |
| --- | --- |
| **Methods** | Randomised, laboratory-based experiment (between-subjects design) |
| **Participants** | **Sampling frame:** University medical school pediatrics clinic  **Geographical region:** USA  **Sampling method:** Opportunity - Selected from outpatient waiting room of pediatrics clinic  **Number of participants randomised:** 75  **Number (%) of enrolled participants completing the study:** 75 (100%) 25 Intervention 1, 25 intervention 2, 25 control  **Study completers - Mean age (SD):** 8-11 years  **Study completers - Sex (Female %):** Not reported  **Ethnicity:** Not reported  **Socio-economic status:** Not reported  **Education:** Elementary school  **Baseline alcohol consumption:** N/A  **Executive function:** Not reported  **Inclusion criteria:** Children 8-11 years of age  **Exclusion criteria:** Not reported |
| **Interventions** | **Intervention 1 –**  **Setting:** Laboratory  **Valence:** Pro  **Medium:** Television  **Title:** “M*A*S*H”  **Total duration of presentation:** 5.5 minutes  **Number of portrayals:** Not reported  **Duration of portrayals:** Not reported  **Brands presented:** Not reported  **Type of alcohol presented:** Not reported  **Editing details:** None  **Control -**  **Setting:** Laboratory  **Valence:** Neutral  **Medium:** Television  **Title:** “M*A*S*H”  **Total duration of presentation:** 5.5 minutes  **Number of portrayals:** Not reported  **Duration of portrayals:** Not reported  **Brands presented:** Not reported  **Type of alcohol presented:** Not reported  **Editing details:** Same 5.5 minute section with the drinking scenes edited out  **Additional details -**  **Intervention arms:** *Intervention 1* – M*A*S*H with alcohol scenes; *Control 1* - M*A*S*H with alcohol scenes edited out; *Control 2* – Television not turned on  **Comparisons analysed:** Intervention 1 v Control  **Concurrent intervention components:** None |
| **Outcomes** | **Outcomes reported in study:** Alcoholic beverage selection without purchasing** (**Task:** Hypothetical choice of beverage to serve to photographs of different individuals; **Unit of measurement**: Number of pictures to which alcohol was offered)  **Timing of outcome measurement:** N/A.  **Additional outcomes:** None reported  ** Primary outcome*  *** Secondary Outcome* |
| **Notes** |  |

| **Slater, Rouner, Murphy, Beauvais, Van Leuven, & Rodriguez (1996)** | |
| --- | --- |
| **Methods** | Randomised, school-based experiment (within-subjects design) |
| **Participants** | **Sampling frame:** 2 junior high schools, 1 high school  **Geographical region:** USA, mid-sized western city  **Sampling method:** Opportunity sample (Mail and classroom solicitation)  **Number of participants randomised:** 157  **Number (%) of enrolled participants completing the study:** 157 (100%)  **Study completers - Mean age (SD):** 14.45 (not reported)  **Study completers - Sex (Female %):** 0  **Ethnicity:** 100% White  **Socio-economic status:** "Lower to upper middle class"  **Education:** Junior high (N = 83); High school (N = 74)  **Baseline alcohol consumption:** N/A  **Executive function:** Not reported  **Inclusion criteria:** Male  **Exclusion criteria:** Not reported |
| **Interventions** | **Intervention 1 –**  **Setting:** Not reported  **Valence:** Pro (with sports content)  **Medium:** Television (Advertisements)  **Title:** Not reported  **Total duration of presentation:** 20 minutes  **Number of portrayals:** 1  **Duration of portrayals:** 0.5 minutes  **Brands presented:** Not reported  **Type of alcohol presented:** Beer  **Editing details:** Beer adverts contained ≥ 8 seconds of beer related content; ad selected from a pool of 24 (within this condition)  **Intervention 2 –**  **Setting:** Not reported  **Valence:** Pro (without sports content)  **Medium:** Television (Advertisements)  **Title:** Not reported  **Total duration of presentation:** 20 minutes  **Number of portrayals:** 1  **Duration of portrayals:** 0.5 minutes  **Brands presented:** Not reported  **Type of alcohol presented:** Beer  **Editing details:** Beer adverts contained ≥ 8 seconds of beer related content; ad selected from a pool of 24 (within this condition)  **Control -**  **Setting:** Not reported  **Valence:** Neutral  **Medium:** Television (Advertisements)  **Title:** Not reported  **Total duration of presentation:** 20 minutes  **Number of portrayals:** 1  **Duration of portrayals:** 0.5 minutes  **Brands presented:** Not reported  **Type of alcohol presented:** N/A  **Editing details:** ad selected from a pool of 24 (within this condition)  **Additional details -**  **Intervention arms:** *Intervention 1* – Beer ad with sports content; *Intervention 2* – Beer ad without sports content; *Control* – Neutral ad  **Comparisons analysed:** Intervention 1 v Control; Intervention 2 v control; Intervention 1 v Intervention 2  **Concurrent intervention components:** None |
| **Outcomes** | **Outcomes reported in study:** Explicit alcohol-related cognitions** (**Task:** Thought listing procedure; **Unit of measurement:** Responses coded into overall response polarity - difference between number of positive and negative thoughts)  **Timing of outcome measurement:** During the experimental manipulation  **Additional outcomes:** Self reported alcoholic beverage consumption (**Task:** Questionnaire - American drug and alcohol survey [Oetting & Beauvais, 1990]; **Unit of measurement:** 7 items, e.g. *"Have you ever had alcohol to drink"*, *"How old were you when you first got drunk"* response format not reported); Sensation seeking (**Task:** Questionnaire - Adventure seeking subscale of Zuckerman scale [Zuckerman, 1979] e.g. *"I sometimes like to do things that are a little bit frightening"*, *"I often wish I could be a mountain climber"*; **Unit of measurement:** Not reported); Masculinity (**Task:** Positive masculine items of Bem sex-role inventory [Bem, 1974] e.g. *"Forceful", "Competitive"*; **Unit of measurement:** Not reported); Sports orientation (**Task:** Questionnaire describing active and spectator roles in sport e.g. *"How active are you in organised sports in the school and community"*, *"How often do you read the sports page in the newspaper?"*; **Unit of measurement:** 8 items, response format not reported)  ** Primary outcome*  *** Secondary Outcome* |
| **Notes** |  |

| **Sobell, Sobell, Riley, Klajner, Leo, Pavan, & Cancilla (1986)** | |
| --- | --- |
| **Methods** | Randomised, laboratory-based experiment (between-subjects design) |
| **Participants** | **Sampling frame:** University of Toronto  **Geographical region:** Canada  **Sampling method:** Opportunity (Posters)  **Number of participants randomised:** 117  **Number (%) of enrolled participants completing the study:** 96 (82.1%) 32 Intervention 1, 32 intervention 2, 32 control  **Study completers - Mean age (SD):** 25.55 (3.7)  **Study completers - Sex (Female %):** 0  **Ethnicity:** 84.38% white  **Socio-economic status:** Not reported  **Education:** 100% A-Level (Level 3); Mean 15.62 years of education  **Baseline alcohol consumption:** 3.32 drinks/drinking day; 56.25% heavy drinkers, 21.88 moderate drinkers, 21.88 light drinkers (Quantity frequency classification: Cahalan, Cisin & Crossley, 1996)  **Executive function:** Not reported  **Inclusion criteria:** ≥ 19 years of age; consumption of beer at least once a month; beer accounting for at least 20% of total alcohol intake; Male  **Exclusion criteria:** score ≥ 3 on Michigan Alcohol Screening test (Selzer et al. 1975); blood alcohol level ≥ 0 at start of the session; Individuals on medication; Individuals who have participated in similar previous studies; Those who have consumed food or drink in the previous 2 hours. |
| **Interventions** | **Intervention 1 –**  **Setting:** Laboratory designed to look like a family room  **Valence:** Pro  **Medium:** Television  **Title:** “Dallas”  **Total duration of presentation:** 60 minutes  **Number of portrayals:** 18 (14 drinking or preparing alcohol, 2 verbal references, 2 visual references)  **Duration of portrayals:** Approximately 14 minutes  **Brands presented:** Not reported  **Type of alcohol presented:** Not reported  **Editing details:** None  **Intervention 2 –**  **Setting:** Laboratory designed to look like a television or family room  **Valence:** Pro  **Medium:** Television (Advertisements)  **Title:** Not reported  **Total duration of presentation:** Not reported  **Number of portrayals:** 4  **Duration of portrayals:** Not reported  **Brands presented:** Not reported  **Type of alcohol presented:** Beer  **Editing details:** Four groups of advertisement equally interspersed in TV programme, presented in groups of 3 (experimental condition ad always first, followed by two filler ads).  **Control 1 -**  **Setting:** Laboratory designed to look like a television or family room  **Valence:** Neutral  **Medium:** Television  **Title:** “Dallas”  **Total duration of presentation:** 60 minutes  **Number of portrayals:** N/A  **Duration of portrayals:** N/A  **Brands presented:** N/A  **Type of alcohol presented:**  **Editing details:** Alcohol scenes edited out and approximately 14 minutes from the beginning of the next episode edited in to give a presentation of similar length.  **Control 2 -**  **Setting:** Laboratory designed to look like a television or family room  **Valence:** Neutral  **Medium:** Television (advertisements)  **Title:** Not reported  **Total duration of presentation:**  **Number of portrayals:** 4  **Duration of portrayals:** Not reported  **Brands presented:** Not reported  **Type of alcohol presented:** N/A  **Editing details:** Four groups of advertisement equally interspersed in TV programme, presented in groups of 3 (experimental condition ad always first, followed by two filler ads).  **Additional details -**  **Intervention arms:** *Intervention 1* – Television programme with alcohol scenes; *Control 1* – Television programme without alcohol scenes; *Intervention* 2 – Alcohol related advertisements; *Control* 2 – Non-alcoholic beverage advertisements; *Intervention 3* – Food related advertisements  **Comparisons analysed:** Intervention 1 v Control 1; Intervention 2 v Control 2; Intervention 1 v Intervention 2  **Concurrent intervention components:** None |
| **Outcomes** | **Outcomes reported in study:** Alcoholic beverage consumption* (**Task:** Beer "Taste test" [Marlatt, 1978]; **Unit of measurement:** ml)  **Timing of outcome measurement:** Immediately following experimental manipulation  **Additional outcomes:** Number of adjective ratings (**Task:** Beer "Taste test" ; **Unit of measurement:** Number of items completed in the taste test)  ** Primary outcome*  *** Secondary Outcome* |
| **Notes** |  |

| **Sumarta (2000)** | |
| --- | --- |
| **Methods** | Randomised, laboratory-based experiment (between-subjects design) |
| **Participants** | **Sampling frame:** University of California  **Geographical region:** California, USA  **Sampling method:** Opportunity (Signed up via bulletin board notice)  **Number of participants randomised:** 144  **Number (%) of enrolled participants completing the study:** 96 (67%) 48 Intervention 1, 48 control  **Study completers - Mean age (SD):** 22.39 (2.96)  **Study completers - Sex (Female %):** 50  **Ethnicity:** 27 Caucasian, 2 African-American, 33 Asian, 22 Hispanic, 12 other  **Socio-economic status:** Not reported  **Education:** A-Level (Level 3) - Inferred from undergraduate student status  **Baseline alcohol consumption:** 21 heavy drinkers, 28 moderate drinkers, 33 light drinkers, 14 infrequent drinkers (Cahalan QF index)  **Executive function:** Not reported  **Inclusion criteria:** Age ≥ 21 years  **Exclusion criteria:** Alcohol abstainers, individuals with previous alcohol or drug problems  144 undergraduate students (48 intervention, 48 control) age: M = 22.39; 50% female |
| **Interventions** | **Intervention 1 –**  **Setting:** University Laboratory with refrigerators, chairs and beer posters  **Valence:** Pro  **Medium:** Television  **Title:** “Cheers”  **Total duration of presentation:** 3.5 minutes  **Number of portrayals:** Not reported  **Duration of portrayals:** Not reported  **Brands presented:** Not reported  **Type of alcohol presented:** Not reported  **Editing details:** Not reported  **Control -**  **Setting:** University Laboratory with refrigerators, chairs and beer posters  **Valence:** Neutral  **Medium:** Television  **Title:** “Newhart”  **Total duration of presentation:** 3.5 minutes  **Number of portrayals:** Not reported  **Duration of portrayals:** Not reported  **Brands presented:** Not reported  **Type of alcohol presented:** Not reported  **Editing details:** Not reported  **Additional details -**  **Intervention arms:** *Intervention 1* – Alcohol related TV programme; *Control* - Neutral TV programme  **Comparisons analysed:** Intervention 1 v Control  **Concurrent intervention components:** None |
| **Outcomes** | **Outcomes reported in study:** Alcoholic beverage consumption* (**Task:** Beer "Taste test" [Marlatt, 1978; **Unit of measurement:** ml [log transformed])  **Timing of outcome measurement:** Immediately following experimental manipulation  **Additional outcomes:** Self reported alcoholic beverage consumption (**Task:** Quantity, Frequency and Variability index [Cahalan, Cisley & Crossley, 1969]; **Unit of measurement:** Quantity of alcohol consumed in each episode, Frequency of consumption episodes); Video Manipulation Evaluation (**Task:** Questionnaire assessing participants ratings of the videos; **Unit of measurement:** Six semantic differential scales "Good/bad", "Beautiful/Ugly", "Pleasant/Unpleasant", "Clear/hazy", "fresh/stale", "fast/slow"); Beverage preference (**Task:** Rating of six drinks in the "taste test"; **Unit of measurement:** Ranking)  ** Primary outcome*  *** Secondary Outcome* |
| **Notes** |  |

| **Van Hoof, de Jong, Fennis, & Gosselt (2009)** | |
| --- | --- |
| **Methods** | Randomised, school-based experiment (between-subjects design) |
| **Participants** | **Geographical region:** Netherlands  **Sampling frame:** Secondary school students  **Sampling method:** “school’s headmaster helped select 12 groups of pupils (three times four groups matched on similar study tracks and years)”  **Number of participants randomised:** 248  **Number (%) of enrolled participants completing the study:** 223 (89.9%)  **Study completers - Mean age (SD):** 14.9 (SD not reported)  **Study completers - Sex (Female %):** 60  **Ethnicity:** Not reported  **Socio-economic status:** Not reported  **Education:** Still in secondary school  **Baseline alcohol consumption:** Not reported  **Executive function:** Not reported  **Inclusion criteria:** Not reported  **Exclusion criteria:** Participants with incomplete forms and extreme outlier scores |
| **Interventions** | **Intervention 1 –**  **Setting:** In school in groups of ~20  **Valence:** Pro  **Medium:** Advertisements (television)  **Title:** N/A  **Total duration of presentation:** Not reported  **Number of portrayals:** 6  **Duration of portrayals:** Not reported  **Brands presented:** Not reported  **Type of alcohol presented:** Beer, spirits, mixed drinks  **Editing details:** Presented in two blocks of ads within a 22 minute episode of a soap opera  **Control -**  **Setting:** In school in groups of ~20  **Valence:** Neutral  **Medium:** Advertisements (television)  **Title:** N/A  **Total duration of presentation:** Not reported  **Number of portrayals:** 6  **Duration of portrayals:** Not reported  **Brands presented:** Not reported  **Type of alcohol presented:** N/A (lemonade advertisements)  **Editing details:** Presented in two blocks of ads within a 22 minute episode of a soap opera  **Additional details -**  **Intervention arms:** *Intervention 1* – Alcohol advertising; *Control* – Neutral advertising  **Comparisons analysed:** Intervention 1 v Control  **Concurrent intervention components:** None |
| **Outcomes** | **Outcomes reported in study:** Explicit alcohol-related cognitions** - Outcome expectancies (**Task:** Questionnaire assessing perceived positive and negative consequences of drinking, **Unit of measurement:** Eleven items with 5-point response scale);  Implicit alcohol-related cognitions** (**Task:** Word completion task, **Unit of measurement:** Mean alcohol references in six target words;  Alcohol selection without purchasing** (**Task:** Hypothetical choice of beverage from five alcoholic or ten non-alcoholic options, **Unit of measurement:** Dichotomous (alcohol/non-alcohol)  **Timing of outcome measurement:** Immediately following manipulation  **Additional outcomes:** Postponed intention to use alcohol – “participants were asked to estimate how many glasses of alcohol they would consume in the next weekend”  ** Primary outcome*  *** Secondary Outcome* |
| **Notes** |  |

| **Wilks, Vardanega, & Callan (1992)** | |
| --- | --- |
| **Methods** | Randomised, laboratory-based experiment (between-subjects design) |
| **Participants** | **Sampling frame:** University of Queensland  **Geographical region:** Brisbane, Australia  **Sampling method:** Opportunity (University research participation scheme)  **Number of participants randomised:** 120  **Number (%) of enrolled participants completing the study:** Not reported  **Study completers - Mean age (SD):** 18-20 years (Mean not reported)  **Study completers - Sex (Female %):** Not reported  **Ethnicity:** Not reported  **Socio-economic status:** Not reported  **Education:** A-Level (Level 3) - Inferred from undergraduate student status  **Baseline alcohol consumption:** Not reported  **Executive function:** Not reported  **Inclusion criteria:** Legal age to buy/consume alcohol  **Exclusion criteria:** Not reported |
| **Interventions** | **Intervention 1 –**  **Setting:** Experimental room with tables, chairs and VHS recorder  **Valence:** Pro  **Medium:** Television (advertisements)  **Title:** Not reported  **Total duration of presentation:** 90 minutes  **Number of portrayals:** 12  **Duration of portrayals:** Not reported  **Brands presented:** Not reported  **Type of alcohol presented:** Not reported  **Editing details:** 90 minutes consisted of 30 minutes of “Neighbours”, 30 minutes of “Hey Dad” and 30 minutes of “World Wide of Sports”; Four adverts were viewed every 30 minutes  **Intervention 2** –  **Setting:** Experimental room with tables, chairs and VHS recorder  **Valence:** Pro  **Medium:** N/A  **Title:** Not reported  **Total duration of presentation:** 90 minutes  **Number of portrayals:** 6  **Duration of portrayals:** Not reported  **Brands presented:** Not reported  **Type of alcohol presented:** Not reported  **Editing details:** 90 minutes consisted of 30 minutes of “Neighbours”, 30 minutes of “Hey Dad” and 30 minutes of “World Wide of Sports”; Two adverts were viewed every 30 minutes  **Control -**  **Setting:** Experimental room with tables, chairs and VHS recorder  **Valence:** Neutral  **Medium:** Television (Advertisements)  **Title:** Not reported  **Total duration of presentation:** 90 minutes  **Number of portrayals:** 12  **Duration of portrayals:** Not reported  **Brands presented:** Not reported  **Type of alcohol presented:** Not reported  **Editing details:** 90 minutes consisted of 30 minutes of “Neighbours”, 30 minutes of “Hey Dad” and 30 minutes of “World Wide of Sports”; Neutral adverts shown - Total number of adverts same as above conditions  **Additional details -**  **Intervention arms:** *Intervention 1* – 12 alcohol advertisements ; *Intervention 2* – 6 alcohol advertisements; *Control* – 0 alcohol advertisements  **Comparisons analysed:** Intervention 1 v Control  **Concurrent intervention components:** None |
| **Outcomes** | **Outcomes reported in study:** Alcoholic beverage consumption* (**Task:** Alcohol consumption during TV viewing, **Unit of measurement:** Number of standard alcoholic drinks consumed);  **Timing of outcome measurement:** During experimental manipulation.  **Additional outcomes:** Alcoholic beverage consumption (**Task:** Questionnaire - Khavari Alcohol Test [Khavari & Farber, 1978]; **Unit of measurement:** Daily average of alcohol consumption (g))  ** Primary outcome*  *** Secondary Outcome* |
| **Notes** |  |

| **Zwarun, Linz, Metzger, & Kunkel (2006)** | |
| --- | --- |
| **Methods** | Randomised, laboratory-based experiment (between-subjects design) |
| **Participants** | **Sampling frame:** Large West Coast University  **Geographical region:** USA  **Sampling method:** Opportunity (Introductory session, for course credit)  **Number of participants randomised:** 215  **Number (%) of enrolled participants completing the study:** 215 (100%) 107 Intervention 1, 108 control  **Study completers - Mean age (SD):** Not reported (34.8% freshmen; 33.1% sophomores; 31.5% juniors, 1 senior)  **Study completers - Sex (Female %):** 80.5%  **Ethnicity:** Not reported  **Socio-economic status:** Not reported  **Education:** A-Level (Level 3) - Inferred from undergraduate student status  **Baseline alcohol consumption:** Not reported  **Executive function:** Not reported  **Inclusion criteria:** Not reported  **Exclusion criteria:** Not reported |
| **Interventions** | **Intervention 1 –**  **Setting:** Laboratory  **Valence:** Pro  **Medium:** Television (Advertisements)  **Title:** Not reported  **Total duration of presentation:** 20 minutes  **Number of portrayals:** Not reported  **Duration of portrayals:** Not reported  **Brands presented:** Bud light, Miller, Molson Ice  **Type of alcohol presented:** Beer  **Editing details:** Not reported  **Control -**  **Setting:** Laboratory  **Valence:** Neutral  **Medium:** Television (Advertisements)  **Title:** Not reported  **Total duration of presentation:** 20 minutes  **Number of portrayals:** Not reported  **Duration of portrayals:** Not reported  **Brands presented:** Not reported  **Type of alcohol presented:** Beer  **Editing details:** Not reported  **Additional details -**  **Intervention arms:** *Intervention 1* – Alcohol advertisements; *Control* – Neutral advertisements  **Comparisons analysed:** Intervention 1 v Control  **Concurrent intervention components:** None |
| **Outcomes** | **Outcomes reported in study:** Explicit alcohol-related cognitions** - Alcohol expectancies (**Task:** Questionnaire - Assessing social benefits, evaluation of risky behaviour engagement with the manipulation and expectancies about physical abilities after alcohol consumption; **Unit of measurement:** Thirty six items, each with 5-point response scale – responses reduced to four factors using principal components analysis)  **Timing of outcome measurement:** Immediately after experimental manipulation.  **Additional outcomes:** Not reported  ** Primary outcome*  *** Secondary Outcome* |
| **Notes** |  |
