# Supplementary material for: Immediate effects of alcohol marketing communications and media portrayals on consumption and cognition: a systematic review and meta-analysis of experimental studies
Source: BMC Public Health. 2016 Jun 9;16:465. doi: 10.1186/s12889-016-3116-8 (PMC4899920; doi:10.1186/s12889-016-3116-8)
Supplement: Additional file 4: — Risk of bias judgements. (DOCX 31 kb) [file 12889_2016_3116_MOESM4_ESM.docx]

Additional file 4

*Risk of bias judgements*

Engels et al. (2009)

| **Bias** | **Authors’ judgement** | **Support for judgement** |
| --- | --- | --- |
| Random sequence generation (selection bias) | Unclear risk | "All 40 couples were randomly assigned to one of four different movie conditions, resulting in 20 males in each condition" |
| Allocation concealment (selection bias) | Unclear risk | Not reported |
| Blinding of participants and personnel (performance bias) | Unclear risk | Not reported |
| Blinding of outcome assessment (detection bias) | Unclear risk | Not reported |
| Incomplete outcome data (attrition bias) | Low risk | Tables indicate N = 20 per condition |
| Selective reporting (reporting bias) | Low risk | All key outcomes measured in the method section are analysed in the results |
| Baseline differences between groups (other bias) | Low risk | "Even though the friendship pairs were randomly assigned to one of the experimental conditions, differences were found on initial drinking habits between the participants in the different conditions"… "Because differences existed in weekly drinking between conditions, we corrected for these differences by conducting an ANCOVA" |
| Consistency of intervention delivery (Other bias) | Unclear risk | "They were told that they would see a movie clip interrupted by two commercial break breaks and just had to act like they were relaxing at home" - Not clear enough indication of a script |
| Validity and reliability of outcome | Unclear risk | No assessment of the correlation between weekly drinking and consumption |

Kohn & Smart (1984)

| **Bias** | **Authors’ judgement** | **Support for judgement** |
| --- | --- | --- |
| Random sequence generation (selection bias) | Low risk | Information received from study author: "The treatment assignment procedure used was as follows: 1) Sessions were scheduled and the scheduled slots were assigned randomly to treatment conditions within subsets of the number of treatments (six for Kohn and Smart (1984) and three for Kohn and Smart (1987)). (The reason for subsetting is that it prevents the possibility of long runs of sessions of any treatment condition which could occur with non-subsetted simple random assignment and would produce a potential confound between treatment and time of running.) 2) Then, participants were assigned to treatments in order of recruitment which was essentially random." |
| Allocation concealment (selection bias) | High risk | Alternation |
| Blinding of participants and personnel (performance bias) | Unclear risk | "The study was represented to subjects as an evaluation of the potential televiewing appeal of indoor soccer." "Every attempt was made to render the deception plausible".  No indication if blinding was successful. |
| Blinding of outcome assessment (detection bias) | Unclear risk | Unclear |
| Incomplete outcome data (attrition bias) | Low risk | All data included in the analyses |
| Selective reporting (reporting bias) | Low risk | All key outcomes measured in the method section are analysed in the results |
| Baseline differences between groups (other bias) | Unclear risk | Not reported |
| Consistency of intervention delivery (Other bias) | Low risk | Procedure appears consistent within and between conditions |
| Validity and reliability of outcome | Unclear risk | No assessment of the correlation between weekly drinking and consumption |

Kohn & Smart (1987)

| **Bias** | **Authors’ judgement** | **Support for judgement** |
| --- | --- | --- |
| Random sequence generation (selection bias) | Low risk | Information received from study author: "The treatment assignment procedure used was as follows: 1) Sessions were scheduled and the scheduled slots were assigned randomly to treatment conditions within subsets of the number of treatments (six for Kohn and Smart (1984) and three for Kohn and Smart (1987)). (The reason for subsetting is that it prevents the possibility of long runs of sessions of any treatment condition which could occur with non-subsetted simple random assignment and would produce a potential confound between treatment and time of running.) 2) Then, participants were assigned to treatments in order of recruitment which was essentially random." |
| Allocation concealment (selection bias) | Unclear risk | Not reported |
| Blinding of participants and personnel (performance bias) | Low risk | Respondents classified on the basis of an awareness questionnaire: "1) What was the purpose of the procedures you underwhent? 2) How did you arrive at your conclusion(s)?" People suspicious about the nature of the study did not significantly differ from those who were not. |
| Blinding of outcome assessment (detection bias) | Unclear risk | Not reported |
| Incomplete outcome data (attrition bias) | Low risk | "Separate analyses were conducted for all subjects (N = 66) and for clearly non-suspicious subjects (N = 54). We also computed an overall two-way ANOVA for treatment condition x suspiciousness" |
| Selective reporting (reporting bias) | Low risk | All key outcomes measured in the method section are analysed in the results |
| Baseline differences between groups (other bias) | Unclear risk | Not reported |
| Consistency of intervention delivery (Other bias) | Low risk | "A female research assistant greeted subjects…She stated that the project concerned the televiewing tastes of college women, an understudied topic with considerable entertainment and commercial implications" |
| Validity and reliability of outcome | Unclear risk | No assessment of the association between weekly drinking and consumption |

Koordeman, Anschutz, & Engels (2011)

| **Bias** | **Authors’**  **judgement** | **Support for judgement** |
| --- | --- | --- |
| Random sequence generation (selection bias) | Unclear risk | Not reported |
| Allocation concealment (selection bias) | Unclear risk | Not reported |
| Blinding of participants and personnel (performance bias) | Low risk | "A naturalistic setting was used to increase the ecological validity of the study and minimize demand characteristics".  “In addition, we asked participants if they became aware of the real aim of the study. Only five participants indicated that they had more or less understood the aim of the study; the others did not think alcohol was involved in the study objectives. We conducted analyses with and without the participants who understood the aim of the study and found the same results.” |
| Blinding of outcome assessment (detection bias) | Unclear risk | Not reported |
| Incomplete outcome data (attrition bias) | Unclear risk | Not reported |
| Selective reporting (reporting bias) | Low risk | All key outcomes measured in the method section are analysed in the results |
| Baseline differences between groups (other bias) | Low risk | "ANOVAs showed no differences between the commercial conditions on age, self-reported weekly alcohol consumption, drinking before the commercials, attitude toward the movie and commercials, and transportation into the movie. A chi- square analysis showed that there was no difference between the commercial conditions on gender, which indicated that group randomization was successful." |
| Consistency of intervention delivery (Other bias) | Low risk | "Participants were allowed to choose a seat in the movie theater. Next, as a cover story, they were told that the researchers were interested in their viewing behaviour. Participants learned that they would watch a movie, and they were asked to act as they normally would in a theater." |
| Validity and reliability of outcome | Low risk | "Higher weekly alcohol consumption was associated with higher levels of alcohol consumption while watching the movie." |

Koordeman, Anschutz, van Baaren, & Engels (2011)

| **Bias** | **Authors’ judgement** | **Support for judgement** |
| --- | --- | --- |
| Random sequence generation (selection bias) | Unclear risk | "A randomised two (sex) x two (movie condition: alcohol, non-alcohol) between subject design was used.” |
| Allocation concealment (selection bias) | Unclear risk | "Participants learned that they would watch a movie for 1 hour and were asked to act as if they were at home" |
| Blinding of participants and personnel (performance bias) | Unclear risk | Not reported |
| Blinding of outcome assessment (detection bias) | High risk | "The researcher coded the amount of bottles and the amount of centilitres consumed and subtracted what was left in the bottle after the session ended" |
| Incomplete outcome data (attrition bias) | Unclear risk | "Participants were 122 same sex dyads of college students" (N = 244) But degrees of freedom show incomplete data "Male participants reported a higher weekly consumption (t [233] = 7.22, p < .001) and scored higher on problem drinking than women (t [240] = 3.21, p = .001)". |
| Selective reporting (reporting bias) | Low risk | All key outcomes measured in the method section are analysed in the results |
| Baseline differences between groups (other bias) | Low risk | "Analyses of variance (ANOVAs) showed no differences between the movie conditionson sex, age, self-reported weekly alcohol consumption, problem drinking, attitude towards the movie, time and day of the week that the experiment took place and whether they had seen the movie before" |
| Consistency of intervention delivery (Other bias) | Unclear risk | Not reported |
| Validity and reliability of outcome | Low risk | "Pearson's correlations showed that self-reported weekly consumption correlated with alcohol consumption while watching" |

Koordeman et al. (2012)

| **Bias** | **Authors’**  **judgement** | **Support for judgement** |
| --- | --- | --- |
| Random sequence generation (selection bias) | Unclear risk | "This study used a randomized between-subject experimental design to compare 2 advertisement conditions" |
| Allocation concealment (selection bias) | Unclear risk | Not reported |
| Blinding of participants and personnel (performance bias) | Low risk | "As a cover story, participants were told that they participated in a study in which they had to judge different documentaries. We told them that we aimed to investigate their judgments in a naturalistic setting, that we created a living room setting, and that we included advertisement breaks for that purpose. First, to further distract them from the real aim of the study, participants completed a questionnaire containing demographic questions and questions about their viewing behaviour"  “91% did not understand the aim of the study”. |
| Blinding of outcome assessment (detection bias) | High risk | "The researcher observed and coded the drinking behaviour in a separate observation room." |
| Incomplete outcome data (attrition bias) | Low risk | 160 participants were randomised, but only the data from 159 were analysed |
| Selective reporting  (reporting bias) | Low risk | All key outcomes measured in the method section are analysed in the results |
| Baseline differences between groups (other bias) | Low risk | "Randomization checks showed no differences between the 2 advertisement conditions concerning age, self-reported weekly alcohol use, problem drinking, attitude toward Advertisements, skepticism toward advertising, and time and day of the week the experiment took place (p > 0.05)." |
| Consistency of intervention delivery (other bias) | Low risk | "Participants were told that they participated in a study in which they had to judge different documentaries. We told them that we aimed to investigate their judgments in a naturalistic setting, that we created a living room setting, and that we included advertisement breaks for that purpose. First, to further distract them from the real aim of the study, participants completed a questionnaire containing demographic questions and questions about their viewing behaviour. Next, they learned they would watch a movie clip for 1 hour interrupted by 3 commercial breaks." |
| Validity and reliability of outcome (other bias) | Low risk | "High weekly drinkers also consumed more in the laboratory" |

Koordeman et al. (2015)

| **Bias** | **Authors’**  **judgement** | **Support for judgement** |
| --- | --- | --- |
| Random sequence generation (selection bias) | Unclear risk | Not reported |
| Allocation concealment (selection bias) | Unclear risk | Not reported |
| Blinding of participants and personnel (performance bias) | Low risk | "As a cover story, participants learned we were interested in their understanding of English movies and that they either watched a movie with Dutch subtitles or a movie without these subtitles and that we would ask questions about the movie afterward."  "Of the participants, 17 indicated they had more or less understood the aim of the study. When conducting the analysis with and without these participants, the findings did not change." |
| Blinding of outcome assessment (detection bias) | High risk | "The researcher observed and coded the drinking behaviour in a separate observation room." |
| Incomplete outcome data (attrition bias) | Unclear risk | Not reported |
| Selective reporting  (reporting bias) | Low risk | All key outcomes measured in the method section are analysed in the results |
| Baseline differences between groups (other bias) | Low risk | "Randomization checks showed there were no differences between the two movie conditions concerning age, whether they had seen the movie before, evaluation of the movie, self-reported weekly alcohol use, binge drinking and problem drinking (p>0.05), indicating that randomization was successful." |
| Consistency of intervention delivery (other bias) | Low risk | "First, participants completed a questionnaire containing demographic questions and questions about their understanding of English to distract them from the real aim of the study. Next they were told that they would watch a movie for 1 hr."  "The alcohol and non-alcohol movie versions were carefully edited in such a way that they did not differ in length and storyline. The same scenes in both conditions were used as often as possible" |
| Validity and reliability of outcome (other bias) | Low risk | Pearson correlation between outcome and self-reported weekly drinking was *r* = .46 |

Roehrich & Goldman (1995)

| **Bias** | **Authors’**  **judgement** | **Support for judgement** |
| --- | --- | --- |
| Random sequence generation (selection bias) | Unclear risk | Participants were randomly assigned into one of four conditions |
| Allocation concealment (selection bias) | Unclear risk | Not reported |
| Blinding of participants and personnel (performance bias) | Unclear risk | Not reported |
| Blinding of outcome assessment (detection bias) | Low risk | "The use of separate experimenters reduced experimenter demand and insured that the experimenter conducting the taste-test survey (the dependent variable) was blind with regard to each participant's status on the two independent variables (type of words and videotapes)." |
| Incomplete outcome data (attrition bias) | Low risk | "Of the 94 women who began the memory study, 80 completed both the memory and consumer survey experiments… The typical participant most frequently drank with friends at parties or in bars. Of the 14 women who did not complete the consumer survey, 2 women declined due to lack of interest in points for participation, 4 women reported allergies to ingredients contained in beer, 2 women were recovering alcoholics, 2 women offered religious proscriptions, 2 women were pregnant, 1 Asian woman feared a flushing response, and 1 woman admitted that she was under 21." |
| Selective reporting (reporting bias) | Low risk | All key outcomes measured in the method section are analysed in the results |
| Baseline differences between groups (other bias) | Low risk | "The groups did not differ as to age (M = 25, 25, 26, and 25), number of drinking episodes each month (M = 5.7, 6.9,5.0, and 4.7), modal number of drinks consumed per occasion (1-2 for each group), or the maximum number of drinks consumed per occasion (M = 5.0, 5.1, 4.4, 5.0)." |
| Consistency of intervention delivery (Other bias) | Low risk | "Four experimenters were thoroughly trained (with a script) to conduct the memory study and were used on a rotating basis; the survey study was conducted by a fifth experimenter." |
| Validity and reliability of outcome | Unclear risk | No assessment of the correlation between weekly drinking and consumption |

Sobell et al. (1986)

| **Bias** | **Authors’ judgement** | **Support for judgement** |
| --- | --- | --- |
| Random sequence generation (selection bias) | Unclear risk | Not reported |
| Allocation concealment (selection bias) | Unclear risk | Not reported |
| Blinding of participants and personnel (performance bias) | Low risk | Efforts made to make taste test appear to be a separate study (e.g. different consent form).  Data from participants who guessed the purpose o the study in a post-experiment questionnaire were excluded from analysis |
| Blinding of outcome assessment (detection bias) | Unclear risk | Unclear if different experimenters were used for the two parts of the study. |
| Incomplete outcome data (attrition bias) | Low risk | 21 additional participants excluded, 14 for guessing the hypothesis or that the two parts of the study were related. No sig diffs on outcomes between these and included participants. Of remaining exclusiong, 6 were for failing to perform adjective task as instructed, 1 was for learning about study from a friend. Differences between these and included participants not analysed. |
| Selective reporting (reporting bias) | Low risk | All key outcomes measured in the method section are analysed in the results |
| Baseline differences between groups (other bias) | Low risk | ANOVAs and chi squared tests showed no differences between groups on demographic and drinking behaviour variables |
| Consistency of intervention delivery (Other bias) | Unclear risk | Order of presentation of certain measures changed half way throughout testing due to participants guessing the purpose of the study. |
| Validity and reliability of outcome | Unclear risk | No assessment of the correlation between weekly drinking and consumption |

Sumarta (2000)

| **Bias** | **Authors’ judgement** | **Support for judgement** |
| --- | --- | --- |
| Random sequence generation (selection bias) | Low risk | "Forty-eight tickets were made for female participants. Each ticket was printed in 1 of 8 possible colors to represent the eight experimental 41 groups. An equal number of tickets were printed in each color. Each ticket was then selected randomly from a container to determine how participants will be randomly assigned to the eight experimental groups." |
| Allocation concealment (selection bias) | Low risk | "A participant's group assignment was decided before his/her actual arrival at the laboratory because the experimenter in the consumer survey was blind to the participant's group assignment in the memory study." |
| Blinding of participants and personnel (performance bias) | Low risk | "A participant's group assignment was decided before his/her actual arrival at the laboratory because the experimenter in the consumer survey was blind to the participant's group assignment in the memory study."  "None of the participants guessed the actual connections between the two studies" (as reported on an experimental experience questionnaire). |
| Blinding of outcome assessment (detection bias) | Low risk | "There was no association between the experimenter's guesses of participants' priming group assignments, correct or incorrect, and the beverage participants were assigned to rate, collapsed over priming groups, *p* > .05." |
| Incomplete outcome data (attrition bias) | High risk | "The number of the participants who dropped out of the Cheers/alcohol expectancy words, Cheers/control words, Newhart/alcohol expectancy words, and Newhart/control words group was 4, 9, 16, and 19 respectively. The five participants whose data was excluded from the consumer survey are included in the attrition analyses. There was an association between the video/word priming groups (4 groups) x study Status (completed or dropped out) chi-square analyses, N = 144) = 8.20, *p* = .04." |
| Selective reporting (reporting bias) | Low risk | All key outcomes measured in the method section are analysed in the results |
| Baseline differences between groups (other bias) | Low risk | No difference between groups on family history for alcoholism or on typical drinking style. |
| Consistency of intervention delivery (Other bias) | Low risk | "All verbal instructions used in the study were read from a script to ensure consistency across all experimenters" |
| Validity and reliability of outcome | Low risk | "Both covariates, participants' QV class and beverage preference, have a linear relationship with the amount of beverage consumed in the taste ratings task" |

Wilks et al. (1992)

| **Bias** | **Authors’ judgement** | **Support for judgement** |
| --- | --- | --- |
| Random sequence generation (selection bias) | High risk | States that participants "were randomly assigned to one of 12 conditions". This is inaccurate, as two conditions (sex and drinker status) were predetermined. Also states that "groups of good friends were limited to only two or three in each condition" suggesting non-random assignment. |
| Allocation concealment (selection bias) | Unclear risk | Not reported |
| Blinding of participants and personnel (performance bias) | Unclear risk | States that "most subjects knew each other" - possibility that participants were aware of other conditions prior to participation |
| Blinding of outcome assessment (detection bias) | High risk | Outcome was assessed by the second author who was in the testing room with the participants during the procedure. |
| Incomplete outcome data (attrition bias) | Low risk | Data from 7.5% (9) of participants were excluded from the analyses on the basis of suspicion about the true nature of the study (assessed using manipulation check questions) |
| Selective reporting (reporting bias) | Low risk | All key outcomes measured in the method section are analysed in the results |
| Baseline differences between groups (other bias) | Unclear risk | Groups differed on typical alcohol consumption (light or moderate-to-heavy) as this was an experimental condition. Other baseline characteristics of groups measured but not compared statistically. |
| Consistency of intervention delivery (Other bias) | Low risk | Procedure appears consistent within and between conditions |
| Validity and reliability of outcome | Low risk | "moderate-heavy drinkers… drank more alcohol than light drinkers" |
